# Blm10/PA200‐Activated 20S Proteasomes Promote α‐Synuclein Degradation and Bypass Proteasome Inhibition in Parkinson's Disease Models

**DOI:** 10.1111/acel.70566

**Published:** 2026-05-28

**Authors:** Tariq T. Ali, Anton Zhornyak, Madiha Merghani, Zora Buschenlange, Eri Sakata, Tiago F. Outeiro, Blagovesta Popova, Gerhard H. Braus

**Affiliations:** ^1^ Department of Molecular Microbiology and Genetics Institute of Microbiology and Genetics, University of Göttingen Göttingen Germany; ^2^ Department of Experimental Neurodegeneration, Centre for Biostructural Imaging of Neurodegeneration University Medical Centre Göttingen Göttingen Germany; ^3^ Institute for Auditory Neuroscience University Medical Centre Göttingen Göttingen Germany; ^4^ German Centre for Neurodegenerative Diseases (DZNE) Göttingen Germany; ^5^ Translational and Clinical Research Institute, Faculty of Medical Sciences Newcastle University Newcastle Upon Tyne UK

**Keywords:** 20S proteasome, alpha‐synuclein, autophagy, Parkinson disease, posttranslational modifications, proteasomal chaperones, protein homeostasis, yeast

## Abstract

Protein homeostasis is essential for maintaining normal cellular function. However, protein homeostasis efficiency declines with age, leading to the accumulation of aberrant protein structures associated with neurodegenerative diseases such as Parkinson's disease (PD). PD is characterized by the aggregation of alpha‐synuclein (αSyn) into cytoplasmic inclusions. This process is accompanied by elevated phosphorylation at serine 129 (S129). The accumulation of αSyn into aggregates and their propagation disrupts key proteostasis pathways, including the ubiquitin–proteasome system (UPS) or autophagy, contributing to cellular dysfunction and neuronal death. This study identified the proteasome activator Blm10 and its human ortholog PA200 as modulators of αSyn degradation and toxicity. The conserved Blm10/PA200 protein plays a key role in regulating proteasome activity and assembly. The αSyn expression increases Blm10 protein stability through autophagy inhibition, in a manner dependent on αSyn phosphorylation at S129 in yeast. Overexpression of *BLM10* or *PA200* reduces αSyn aggregation and enhances αSyn turnover via activation of the 20S proteasome in yeast and mammalian cells. Blm10 and PA200‐capped 20S proteasomes efficiently degrade both monomeric as well as oligomeric αSyn in vitro. Notably, capped proteasomes retain proteolytic activities in the presence of αSyn, indicating resistance to αSyn‐induced inhibition, in contrast to 20S or 26S proteasomes. These results reveal a distinct proteasome subtype that bypasses UPS impairment and restores proteolytic capacity under proteotoxic stress. Our findings establish Blm10/PA200 as critical regulators of αSyn proteostasis and highlight its protective role in maintaining protein homeostasis and cell viability under conditions of αSyn toxicity.

## Graphical abstract

This study demonstrates that α‐synuclein expression stabilizes the proteasome activator Blm10, thereby linking α‐synuclein stress to changes in proteasome composition. Blm10/PA200‐capped 20S proteasomes efficiently degrade both monomeric and oligomeric α‐synuclein and remain resistant to α‐synuclein‐induced inhibition. Our findings reveal a proteasome configuration that maintains proteolytic activity under proteotoxic stress.

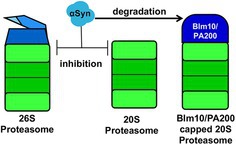

## 1 Introduction

Proteasomal dysfunction is a hallmark of numerous neurodegenerative disorders, including Parkinson's disease (PD). Proteasome activity declines with aging and can lead to the accumulation of misfolded α‐synuclein (αSyn) protein aggregates. During disease progression, αSyn undergoes a conformational shift from a soluble, natively unfolded protein to β‐sheet‐rich oligomers and insoluble amyloid fibrils, which accumulate as Lewy bodies (LB), the pathological hallmark of PD (Mehra et al. 2021; Spillantini et al. 1997; Winner et al. 2011). This aggregation is related to a failure in protein clearance mechanisms, particularly the ubiquitin‐proteasome system (UPS), which itself deteriorates with age (Hipp et al. 2019). Moreover, αSyn has been shown to impair both autophagy (Fellner et al. 2021) and proteasomal degradation (Lindersson et al. 2004; Thibaudeau et al. 2018), creating a pathological feedback loop that exacerbates proteotoxic stress. The precise molecular mechanisms connecting proteasome dysfunction to PD pathology remain poorly understood, although the inhibition of proteasomal activity has been linked to αSyn aggregation and neuronal loss. Elucidating these processes is essential for understanding disease progression and for identifying therapeutic targets within the UPS.

Soluble αSyn is primarily cleared by the UPS, whereas aggregated and oligomeric forms are preferentially degraded through autophagy (Petroi et al. 2012; Webb et al. 2003). Key regulatory determinants of αSyn turnover are its post‐translational modifications, particularly phosphorylation at serine 129 (pS129), which is highly enriched in pathological aggregates (Anderson et al. 2006; Oueslati 2016). This modification influences αSyn aggregation propensity, toxicity, and susceptibility to degradation, making it a focal point in PD pathophysiology (Kleinknecht et al. 2016; Popova et al. 2015; Shahpasandzadeh et al. 2014; Stefanis et al. 2019). The expression of αSyn disrupts proteasome homeostasis in cellular models by reducing the abundance of proteasomal subunits (McNaught et al. 2003; Popova, Galka, et al. 2021) and by impairing the assembly and activity of 26S proteasomes (Galka et al. 2024). αSyn degradation is mediated by different types of proteasomes, each with distinct roles. The 26S proteasome, which requires ATP and targets polyubiquitinated substrates, is primarily responsible for degrading soluble αSyn under normal conditions (Bi et al. 2021).

In contrast, the 20S core particle (CP) can directly degrade unstructured αSyn by ubiquitin‐ and ATP‐independent mechanisms (Tofaris et al. 2001). This pathway is particularly relevant during oxidative stress when ubiquitination is compromised. Oxidative stress has been shown to occur in cells with αSyn pathology (Hsu et al. 2000). Additionally, hybrid proteasomes exist in cells, formed by the association of the 20S core with alternative regulatory particles that possess intermediate substrate specificities (Mayor et al. 2016). These variants could influence αSyn turnover during cellular stress or inflammation; however, their specific contribution is not yet defined. Understanding the balance and regulation among these proteasomal subtypes is critical for elucidating αSyn homeostasis and its disruption in PD.

In our recent high‐throughput screen, we identified the proteasome activator Blm10 as a protein stabilized in the presence of αSyn (Galka et al. 2024), suggesting a potential role in αSyn homeostasis. Yeast Blm10 as well as its human ortholog PA200 bind the 20S proteasome and enhance the degradation of small, unfolded proteins (Dange et al. 2011; Schmidt et al. 2005; Weberruss et al. 2013). Expression levels of the *PSME4* gene which encodes PA200, as well as *BLM10*, decrease during aging, making them potentially interesting targets for analysis in the context of PD (Chen et al. 2020). In this study, we investigate the functional contribution of Blm10 in counteracting αSyn toxicity using *Saccharomyces cerevisiae* as a well‐established reference cell system for PD (Outeiro and Lindquist 2003; Petroi et al. 2012). Elevated Blm10 levels improve cell viability, accelerate αSyn turnover, and restore proteasome activity both in vivo and in vitro. Importantly, the effects observed in yeast proteasomes with Blm10 could be recapitulated by human 20S proteasomes capped with PA200. The findings demonstrate that Blm10/PA200‐mediated 20S proteasome activation enhances αSyn degradation and mitigates proteasome dysfunction. These results reveal that targeting proteasome activation through subunit assembly or 20S gate opening mechanisms has a promising potential as a therapeutic strategy to promote αSyn clearance. Together, these findings uncover a previously unrecognized mechanism linking αSyn stress to proteasome remodeling and provide new insight into how cells adapt protein degradation pathways to proteotoxic stress.

## 2 Experimental Procedures

The yeast strains and plasmids used are listed in Tables S1 and S2.

### 2.1 Yeast Strains, Transformations and Culture Conditions

Plasmids were constructed using GENEART seamless cloning and assembly kit (Invitrogen, USA) and verified by DNA sequencing. *S. cerevisiae* yeast strains were grown at 30°C in YEPD (Yeast‐Extract‐Peptone‐Dextrose) media or in synthetic complete (SC) dropout medium, lacking the respective amino acids for selection. The medium was supplemented with either 2% glucose, 2% raffinose, or 2% galactose. Expression of genes under *GAL1* promotor control was achieved by supplementing the SC medium with 2% galactose. Yeast transformations were performed using the standard lithium acetate procedure (Gietz et al. 1992) and plasmids isolated from DH5α *E. coli* bacteria. Human embryonic kidney 293 (HEK) cells were maintained as described previously (Popova, Wang, et al. 2021).

### 2.2 Fluorescence Microscopy

Overnight cell cultures cultivated in raffinose‐containing SC medium lacking amino acids for selection were diluted to OD_600_ = 0.3 and transferred to SC medium containing 2% galactose. After induction for 6 h, 300 μL cells were transferred into 8‐well Ibidi Dishes (Ibidi, Germany) and imaged using a Zeiss Observer Z1 microscopy (Carl Zeiss, Germany) equipped with a CSU‐X1 A1 confocal scanner unit (Yokokgawa, Japan) and a QuantEM:512SC digital camera (Photometrics, USA). Fluorescence intensities were measured using Slide Book 6.0 software package (Intelligent Imaging Innovations, USA).

### 2.3 Cycloheximide Chase Experiment

Genes coding for target proteins were expressed for 6 h before the cultures were diluted to equal cell density and supplemented with 50 μg/mL cycloheximide to stop translation. An equal volume of samples was collected at time points 0, 1, and 2 h. Cells were pelleted, frozen in liquid nitrogen, and used for crude protein extraction. This approach preserves dilution effects caused by cell growth after translation arrest and allows accurate determination of protein decay kinetics (Khmelinskii et al. 2014).

### 2.4 Immunoblotting

Yeast cells were harvested by centrifugation and yeast protein extraction was performed by NaOH lysis and trichloroacetic acid (TCA) precipitation. The cell pellet was resuspended in 1 mL 0.25 M NaOH with 1.5% 2‐mercaptoethanol for cell lysis. After 15 min incubation on ice, the proteins were precipitated with 150 μL 55% TCA. Protein pellets were resuspended in HU buffer (200 mM Tris–HCl pH 6.8; 8 M Urea; 5% SDS; 1 mM EDTA; 0.05% bromophenol blue) and denatured at 65°C for 10 min. Gel electrophoresis was performed in 9% or 12% SDS‐acrylamide gels, depending on the size of the proteins of interest. Proteins were blotted onto a nitrocellulose membrane (GE Healthcare, USA) and western hybridization analyses were performed using standard procedures (Popova, Wang, et al. 2021). Antibodies used are listed in Table S3. Quantifications of pixel densities were obtained from TIFF files originating from digitized X‐ray films (Cytiva, USA) and analyzed with ImageJ software (NIH, USA).

### 2.5 Spotting Assay

Spotting assays for analysis of yeast growth were performed using cells grown overnight in selective SC medium supplemented with 2% raffinose. Cells were diluted to OD_600_ = 0.1 and serially diluted 10‐fold. Cells were spotted in a volume of 10 μL onto selective SC plates containing glucose as a control or galactose to induce expression of genes under *GAL1* promotor. Plates were incubated at 30°C and documented after 3 days.

### 2.6 Growth Analysis in Liquid Culture

Growth of yeast cells was analyzed in liquid media after overnight growth in selective SC media containing 2% raffinose at 30°C in a clear 96‐well plate in a Tecan Infinite M200 (Tecan, Switzerland) plate reader till all samples reached stationary phase. The next day 1 μL of each sample was transferred onto a new 96‐well plate with selective SC medium containing raffinose. After 90 min, 20% galactose was diluted in the cultures to a concentration of 2% in order to induce expression of genes under *GAL1* promotor. Cell growth at 30°C was observed for 20 h by measuring the absorbance at 600 nm.

### 2.7 Peptidase Activity Measurement

Proteasome activities were measured through the degradation of the fluorogenic peptide SUC‐LLVY‐AMC (Enzo Life Science, USA) and the subsequent release of 7amino‐4‐methylcoumarin. Fluorescence was measured for 30 min at 37°C using Tecan Infinite M200 plate reader (excitation wavelength = 350 nm; emission wavelength = 440 nm). Crude protein extracts were harvested from transformed yeast cells that were grown in selective SC medium containing galactose for 16 h to induce expression of genes under *GAL1* promotor. Yeast cells were lysed by cryo‐milling in buffer A (50 mM Tris–HCl pH 7.4; 100 mM NaCl; 10% Glycerol; 10 mM MgCl_2_; 4 mM ATP). Human epithelial kidney (HEK) cells were lysed by sonication in lysis buffer (40 mM Tris–HCl pH 7.5; 50 mM NaCl;2 mM DTT, 5 mM Mg_2_Cl, 2 mM ATP; 10% glycerol) supplemented with protease inhibitor mix (PIM; Roche Diagnostics, Germany). Cell lysates were cleared by centrifugation at 4°C for 15 min at 15.000×*g*. Concentration of protein extract was determined by Bradford assay and 100 μg crude protein extract were used for 26S activity assays using SUC1‐buffer (20 mM Tris–HCl pH 7.4, 50 mM NaCl, 2 mM DTT, 5 mM MgCl_2_, 2 mM ATP) supplemented with 100 μM SUC‐LLVY‐AMC. The proteasome inhibitor MG132 (MedChemExpress, USA) was used as control. Crude protein extracts were preincubated with 100 μM MG132 for 10 min prior to measurement. Lack of proteasome activity is indicative for the specificity of the assay. To assess the activity of 20S proteasomes in crude protein extracts, 0.025% SDS was added to the SUC1‐buffer. For determination of the activity of in vitro reconstituted 20S + Blm10 or human 20S + PA200, purified 20S proteasomes were reconstituted with the respective proteasomal activator for 30 min at 30°C. To assemble the proteasomes, 250 nM proteasome and 500 nM Blm10 or PA200 were added to the reconstitution buffer (50 mM Tris–HCl pH 7.4; 5 mM MgCl_2_; 0.5 mM EDTA). 0.5 μg purified 20S proteasomes were used for activity assays. The peptidase activity of purified 20S proteasomes was measured using 100 μM SUC‐LLVY‐AMC in SUC2‐buffer (50 mM Tris–HCl pH 7.4, 100 mM NaCl, 10 mM MgCl_2_, 2 mM DTT) as described above.

### 2.8 Protein Purification

Proteasomes were purified using *RPN11‐3xFLAG* and *PRE1‐3xFLAG* strains for 26S and 20S proteasomes, respectively. Cells were grown in YPED media overnight, harvested, and cryo‐milled in buffer A (for 26S) or buffer B (50 mM Tris–HCl pH 7.4; 500 mM NaCl, 1 mM EDTA) for 20S proteasomes. The absence of ATP and increased NaCl concentration removes the regulatory particle from the core particle (Li et al. 2015). Human proteasomes were purchased commercially (Enzo Life Science, USA). Yeast cells transformed with plasmids coding for 3xFLAG‐Blm10 were grown in selective SC media containing galactose to induce gene expression. Cells were harvested and cryo‐milled in buffer B supplemented with protease inhibitor mix (PIM; Roche Diagnostics, Germany). Cell lysates were centrifuged for 30 min at 15,000×*g* at 4°C, and the supernatant was used for FLAG affinity purification using the M2 affinity gel (Merck KGaA, Germany) according to the manufacturer protocol. For the purification of PA200, the protein was genetically fused to a 6xHis tag. The corresponding coding sequence was cloned into a yeast plasmid and expressed in yeast cells. Gene expression was induced with galactose overnight, after which cells were harvested. Cells were cryo‐milled in native binding buffer (50 mM NaH_2_PO_4_; pH 8.0; 500 mM NaCl, 10 mM Imidazole) supplemented with protease inhibitor mix (PIM; Roche Diagnostics, Germany). Cell lysates were cleared by centrifugation at 4°C for 15 min at 15.000×*g*. Native protein purification of His‐tagged PA200 was performed according to the Novex Ni‐NTA manufacturer protocol (Thermo Fisher Scientific, USA) using Ni‐NTA agarose beads (Qiagen N.V., The Netherlands).

### 2.9 Expression and Purification of Human Recombinant αSyn

Expression and purification of αSyn was performed as described previously (Miranda et al. 2013). *E. coli* strain BL21 (DE3) was transformed with pME4913 and the expression was induced in LB medium using 1 mM isopropyl‐β‐D‐1‐thiogalactopyranoside (IPTG). The overnight culture was inoculated at OD_600_ = 0.3, grown at 37°C, pelleted and flash‐frozen at −80°C. Cells were lysed using by sonication (five steps, 30 s per step, 1 min cool down in‐between steps) in *E. coli* lysis buffer (750 mM NaCl, 10 mM Tris pH 8.0, 1 mM EDTA, 1 mM PIM). Afterwards the lysate was heated to 95°C for 15 min and cleared by centrifugation at 4°C for 10 min at 15.000 g. Supernatant was dialyzed overnight at 4°C against the dialysis buffer (50 mM NaCl, 10 mM Tris pH 7.6, 1 mM EDTA). αSyn was purified using HiTrap Q FF 1 mL anion exchange columns (GE Healthcare, USA) using a NaCl gradient from 0 to 600 mM in 25 mM Tris pH 7.7. Fractions were collected and analyzed for αSyn purity using SDS‐PAGE. αSyn monomers were purified using HiLoad 16/600 Superdex 75 prep grade 120 mL column (GE Healthcare). Elution was performed in SEC buffer (25 mM HEPES, 100 mM NaCl,1 mM DTT, pH 8.0). αSyn oligomers were generated by incubating monomeric αSyn in 25 mM Tris (pH 7.7) and 150 mM NaCl overnight at 4°C without agitation. The oligomers were separated from the remaining monomers by size‐exclusion chromatography using the same set‐up. The oligomeric fraction was analyzed by SDS‐PAGE and dot blot using A11 antibody. High‐molecular‐weight αSyn oligomeric species were generated under constant shaking in PBS (pH 7.4) for 20 h at either 20°C or 37°C and analyzed by SDS‐PAGE and Native PAGE.

### 2.10 Native PAGE and In‐Gel Activity

Native PAGE with following in‐gel activity measurement was performed with reconstituted 20S proteasomes. Gel electrophoresis was performed using 4% acrylamide gel complemented with 5 mM MgCl_2_ and 2.5% sucrose and 3% stacking gel. Electrophoresis was run for 3 h on ice at 100 V using filter sterilized native running buffer (90 mM Tris‐Borate pH 8.3; 5 mM MgCl_2_). In‐gel peptidase activity was measured after incubating the gel for 10 min at 37°C using SUC2‐buffer supplemented with 100 μM SUC‐LLVY‐AMC. The resulting fluorescent activity was captured using Fusion FX6 Edge Imaging System (Vilber, France) using the corresponding software. Signal intensity in the resulting TIFF files was measured using Image J (NIH, MD, USA).

### 2.11 Negative Stain Transmission Electron Microscopy

Purified 20S proteasomes were incubated with purified Blm10 at indicated concentrations for 30 min at 30°C. For negative stain analysis, samples were loaded on a continuous carbon film. The copper grid (CF200, Electron Microscopy Sciences, PA, USA) was glow‐discharged using a plasma cleaner (HARRICK PLASMA, USA). The carbon film was attached to the grid and stained in 2% Uranyl‐acetate solution (Science Services) for 1 min. Afterwards, the sample was dried and imaged using Tecnai G2 spirit (Thermo Fisher Scientific, USA).

### 2.12 In Vitro Degradation of αSyn

Purified αSyn protein was incubated with 20S proteasomes or 20S + Blm10 or 20S + PA200 complexes, reconstituted as described above to analyze the proteasomal degradation of αSyn. For each time‐point, 0.5 μg of αSyn was added to 2.4 μg of proteasomes. Degradation of αSyn occurred at 30°C over a 2‐h time frame, and samples were taken at the indicated time points and denatured at 95°C for 10 min in Laemmli buffer (62.5 mM Tris–HCl pH 6.8; 2% SDS; 2.5% β‐mercaptoethanol, 5% glycerol; 0.005% bromophenol blue). Degradation of αSyn was monitored using SDS‐PAGE with subsequent immuno‐hybridization analysis.

### 2.13 Human Cell Culture and Transfection

Human neuroglioma H4 cells were cultured at 37°C and 5% CO_2_ in Opti‐MEM Reduced Serum Medium (Life Technologies‐Ginco, Carlsbad, CA), supplemented with 10% fetal bovine serum (FBS) (PAA, Cölbe, Germany) and 1% Penicillin–Streptomycin. Twenty‐four hours prior to transfection, cells were seeded into 12‐well plates (Costar, Corning, New York). Cells were transfected with FuGENE (Promega, WI, USA) according to the manufacturer's protocol. Plasmids encoding SynT and synphilin‐1 were co‐transfected at a 1:1 ratio, or together with PA200 at a 1:1:1 ratio. Forty‐eight hours post‐transfection, cells were fixed for immunostaining.

### 2.14 Immunocytochemistry

Cells were fixed with 4% paraformaldehyde in DPBS and washed three times. Permeabilization was performed for 10 min with 0.1% Triton X‐100 in DPBS, followed by blocking for 1 h at room temperature with 1.5% normal goat serum (S1000, Vector). Cells were incubated overnight at 4°C with primary antibodies: mouse anti‐α‐synuclein (Syn1, 1:1000; BD Transduction Laboratories) and rabbit anti‐FLAG (1:1000; F‐7425, Sigma‐Aldrich). After PBS washes, cells were incubated for 1 h at room temperature with Alexa Fluor 488 donkey anti‐mouse IgG and Alexa Fluor 555 goat anti‐rabbit IgG (both 1:1000; Invitrogen). Nuclei were stained with DAPI (Carl Roth, Germany) for 5 min. Coverslips were mounted using Fluoromount‐G (Invitrogen) and stored at room temperature until imaging.

### 2.15 Statistical Analysis

Quantitative data were analyzed using GraphPad Prism 6 software (San Diego, USA) and presented as mean ± SEM of at least three individual replicates. Statistical significance was assessed as described in the figure legend, with *p* < 0.05 considered significant.

## 3 Results

### 3.1 αSyn‐Induced Autophagy Impairment Enhances the Stability of the 20S Proteasome Activator Blm10

**FIGURE 1:**
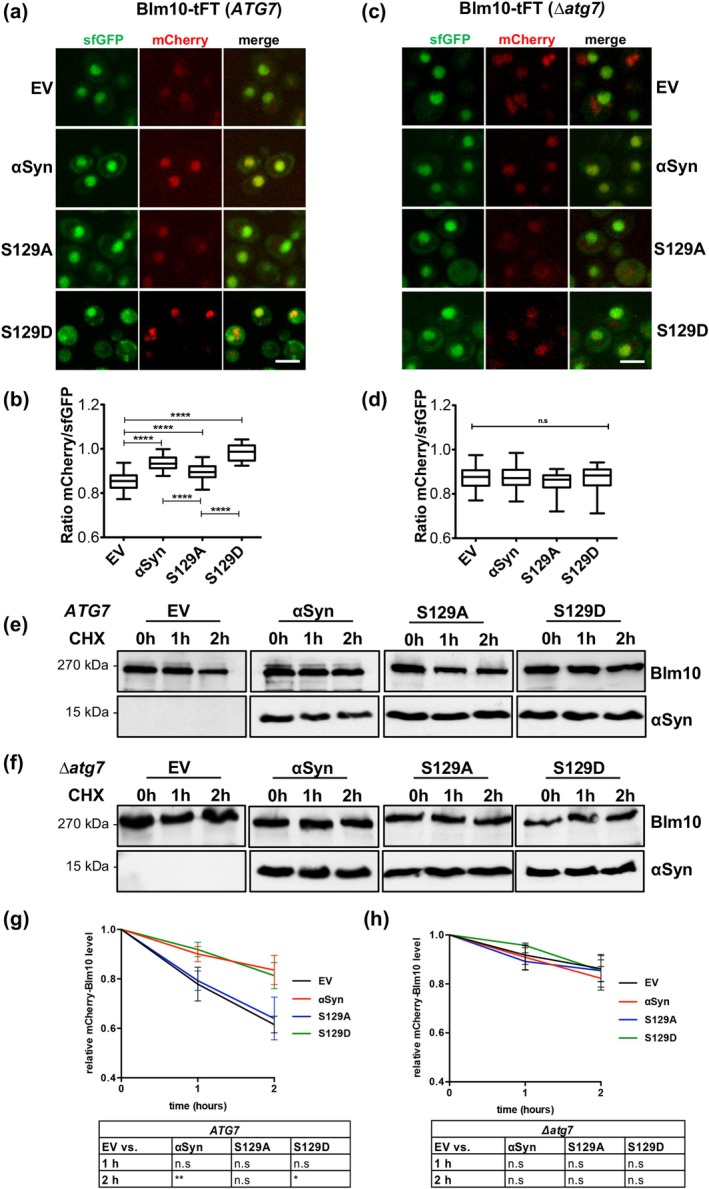
αSyn expression increases Blm10 stability via autophagic inhibition. (a) Tandem Fluorescent Timer (tFT) analysis of Blm10 stability by fluorescence microscopy. Different maturation kinetics of superfolder GFP (sfGFP) (fast) and mCherry (slow) allow estimation of protein stability based on mCherry/sfGFP fluorescence ratios. Expression of αSyn or its variants for 6 h significantly increases Blm10 stability versus empty vector (EV) control. Scale bar = 5 μm. (b) Quantification of single‐cell mCherry/sfGFP ratios from (a). Statistical significance was determined by one‐way ANOVA followed by Dunnett's multiple comparison test (****p < 0.0001; *n* = 50). (c) tFT analysis of Blm10 stability in autophagy‐deficient (*∆atg7*) cells expressing αSyn or its variants. Scale bar = 5 μm. (d) Quantification of mCherry/sfGFP ratios in *∆atg7* cells, as performed in (b), reveals no significant differences in Blm10 stability between EV and αSyn‐expressing conditions. (e, f) Immunoblot analysis of Blm10 degradation after cycloheximide (CHX) chase in autophagy‐competent *ATG7* (e) and autophagy‐deficient *∆atg7* (f) cells. The time post treatment with CHX is indicated above the blots. Blm10 was expressed as N‐terminally tagged mCherry fusion protein. (g, h) Densiometric quantification of Blm10 levels from immunoblots at indicated times post‐CHX treatment in *ATG7* (g) and *∆atg7* (h) cells. Protein levels were compared to Blm10 levels prior to CHX treatment. Statistical analysis was performed using a two‐way ANOVA with Dunnett's multiple comparison test comparing αSyn and its mutants to the EV control (*p < 0.05; **p < 0.01; n.s *p* > 0.05; *n* = 3).

A proteome‐wide screen using tandem fluorescent protein timer (tFT) fusions was previously conducted to explore changes in protein stabilities upon expression of αSyn or the phosphorylation‐deficient S129A mutant in yeast (Galka et al. 2024). tFT construct consists of a tandem fusion of mCherry and superfolder GFP (sfGFP), fluorescent proteins with distinct kinetics of fluorophore maturation. tFT reports on the degradation kinetics of protein of interest fused to the N‐terminus of tFT. Fusions undergoing fast turnover are degraded before mCherry maturation, resulting in a low mCherry/sfGFP intensity ratio, whereas the relative fraction of mature mCherry increases for proteins with slow turnover. This enables direct visualization of protein stability through color changes over time. Expression of αSyn led to significant disruption of protein homeostasis and revealed a novel connection between αSyn and 26S proteasome assembly. Expression of αSyn or the phosphorylation‐deficient S129A variant increased the stability of the proteasomal activator Blm10‐tFT. Blm10 emerged as a top candidate among the stabilized proteins. Live cell fluorescence microscopy of *BLM10*‐tFT yeast strain showed significantly stronger Blm10 stabilization upon αSyn expression compared to S129A (Figure 1a,b). Expression of the S129D variant protein that mimics constant phosphorylation at S129 further confirmed that αSyn phosphorylated at S129 affects Blm10 stability significantly more than the non‐phosphorylatable S129A version. Similarly, hyperphosphorylation of αSyn through overexpression of the human kinase GRK5 produced a comparable effect (Figure S1). This kinase is known to phosphorylate αSyn at S129 in humans as well as in yeast (Shahpasandzadeh et al. 2014). Blm10, like its mammalian ortholog PA200, contains a conserved C‐terminal HbYX motif that promotes 20S proteasome gate opening and enhances proteolytic activity (Chuah et al. 2023). Both Blm10 and PA200 promote ubiquitin‐independent degradation of unfolded, aggregation prone proteins such as tau and huntingtin in vitro (Aladdin et al. 2020), and have been shown to mitigate proteasomal inhibition caused by these proteins (Chuah et al. 2023). Next, the question was addressed, whether the effect of αSyn or S129A on Blm10 stability is mediated by direct physical interaction between the proteins using two different approaches. Yeast‐two‐hybrid (Y2H) (Figure S2a) and Bimolecular fluorescence complementation (BiFC) experiments (Figure S2b,c) revealed that there is no direct interaction between the proteins. These findings suggest that the impact of αSyn on Blm10 stability is likely mediated by indirect effects rather than direct physical interaction.

Under conditions of physiological *BLM10* expression, Blm10 is degraded predominantly by autophagy (Burris et al. 2021). αSyn negatively influences both the selective and nonselective autophagy pathways (Sahoo et al. 2022). Therefore, it was assessed whether Blm10 stabilization by αSyn is due to reduction of its degradation through the autophagy pathway. The impact of αSyn variants on autophagy was tracked using the autophagy marker GFP‐Atg8 which associates with autophagosomal membranes and enables visualization of autophagosomes. Expression of αSyn variants coincided with accumulation of autophagosomes in the cytoplasm, indicating inability of autophagosomes to fuse with the vacuole of yeast cells (Figure S3a,b). Additionally, the impact of αSyn and S129 phosphorylation on autophagy flux was assessed. Autophagy was induced by nitrogen starvation and the cleavage of GFP from GFP‐Atg8 upon delivery to the vacuole was monitored (Figure S3c–e). The results revealed that expression of αSyn inhibits autophagy and that this effect depends on the phosphorylation of S129. The effect of αSyn on Blm10‐tFT stability was further investigated in the *∆atg7* deletion strain. *ATG7* encodes a protein that is required for autophagy as it activates the ubiquitin‐like proteins Atg8 and Atg12, which are indispensable for autophagosome formation. Inability of cells to perform autophagy was verified in the presence of αSyn variants and in control cells (Figure S3f). No difference in Blm10‐tFT stability was observed between the control and αSyn or its variants when autophagy is inhibited (Figure 1c,d), indicating that autophagic degradation is the major regulatory pathway of Blm10 stability affected by αSyn expression.

Cycloheximide chase experiments using N‐terminally tagged mCherry‐Blm10 further confirmed that the change in Blm10 stability is caused by reduced autophagic degradation (Figure 1e–h). Cells harvested at indicated time points were used for immuno‐hybridization and the degradation of Blm10 was followed over time. In cells with intact *ATG7*, expression of αSyn led to increased stability of mCherry‐Blm10 (Figure 1e,g). The inhibition of Blm10 degradation was higher in cells with phosphorylatable S129, corroborating the results from Blm10‐tFT experiments. In the ***∆**atg7* strain, no significant differences in Blm10 degradation were observed (Figure 1f,h). In summary, the presence of αSyn impairs the degradation of Blm10 through inhibition of autophagy. Since the inhibition of autophagy is dependent on the phosphorylation of S129, the increased stability of Blm10 is also linked to the phosphorylation of this residue.

### 3.2 Elevated *BLM10* / *PSME4* Expression Levels Reduce αSyn Aggregation in Yeast and Mammalian Cells and Improve Yeast Cell Growth

**FIGURE 2:**
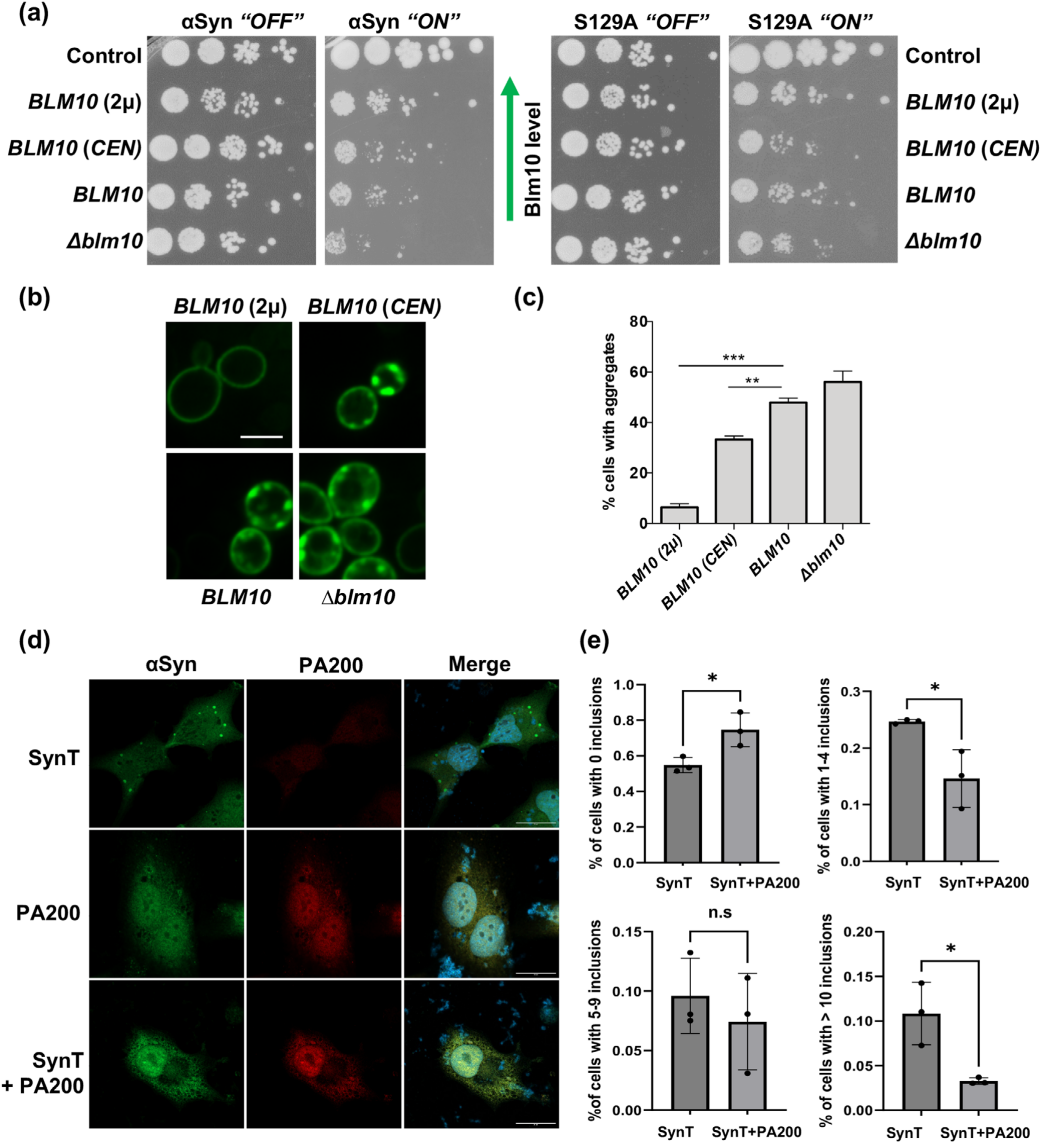
Increased Blm10/PA200 levels reduce αSyn aggregation in yeast and mammalian cells. (a) Yeast growth assays with increasing levels of Blm10. Yeast cells were transformed with either low‐copy (*CEN*) or high‐copy (2 μ) plasmids expressing *BLM10* and co‐transformed with *GAL1* promoter‐driven constructs expressing either wild‐type αSyn (*SNCA*) or its phosphorylation‐deficient mutant S129A. *BLM10* denotes endogenous *BLM10* expression. Control represents wild‐type cells with empty vector. Growth was assessed after 3 days. (b) Fluorescence microscopy of yeast cells expressing αSyn‐GFP and varying levels of *BLM10* after 6 h of *GAL1*‐driven αSyn expression. αSyn‐GFP forms visible cytoplasmic foci, initiating at the plasma membrane. (c) Quantification of αSyn‐GFP foci from (b). Statistical significance was determined using one‐way ANOVA with Dunnett's post hoc test versus endogenous *BLM10* (***p < 0.001; **p < 0.01; *n* = 3). (d) Fluorescence microscopy of human neuroglioma cells transfected with synphilin‐1 and synT, either alone or in combination with PA200. Cells were fixed and immunostained for αSyn and PA200. Scale bar = 30 μm. (e) Quantification of transfected human H4 cells exhibiting cytoplasmic inclusions, comparing endogenous versus overexpressed PA200 conditions. Cells were categorized by inclusion number: no inclusions, 1–4 inclusions, 5–9 inclusions, or > 10 inclusions. Data are presented as mean ± standard deviation. Statistical significance was determined by Student's *t*‐test (*p < 0.05; n.s *p* > 0.05; *N* = 3).

Given the observed increase in Blm10 stability in the presence of αSyn, it was investigated whether elevated levels of Blm10 modulate αSyn‐associated toxicity and aggregation and how pS129 contributes to this process. αSyn and S129A were co‐expressed with mCherry‐*BLM10*, testing different levels of *BLM10* by expression from low‐copy (*CEN*) or high‐copy (2 μ) plasmids. Growth tests were performed to determine αSyn or S129A‐associated cytotoxicity. High‐level overexpression of *BLM10* improved the growth of yeast cells expressing either wild‐type αSyn or the S129A variant (Figure 2). Low‐level overexpression from a *CEN* plasmid conferred minimal or no protective effect compared to endogenous *BLM10* expression. Deletion of *BLM10* did not significantly affect the growth of αSyn‐expressing cells, suggesting that Blm10 may partially rescue αSyn toxicity when expressed at supraphysiological levels. Fluorescence microscopy was used to determine whether Blm10 influences cytotoxicity associated with αSyn‐GFP aggregation. Cells expressing αSyn‐GFP exhibited prominent cytosolic aggregates (Figure 2b,c). In contrast, Blm10‐overexpressing cells showed a significant reduction in αSyn‐GFP aggregation even at moderate overexpression levels.

Next, we assessed whether elevated levels of PA200 inhibit αSyn aggregation in human neuroglioma (H4) cells. αSyn aggregation can be induced by co‐expression of a C‐terminally modified form of αSyn (SynT) and synphilin‐1, an αSyn‐interacting protein that facilitates the formation of intracellular αSyn inclusions (Lázaro et al. 2014). Therefore, H4 cells were co‐transfected with plasmids encoding SynT, synphilin‐1, and PA200. Similar to the findings from yeast cells, overexpression of *PSME4* reduced the percentage of cells with αSyn inclusions as well as the number of inclusions per cell (Figure 2d,e). These results corroborate that elevated levels of Blm10/PA200 rescued αSyn‐associated growth retardation in yeast and reduced αSyn aggregation in yeast and in mammalian cells, respectively. The findings highlight the protective role of Blm10/PA200 in mitigating αSyn aggregation and toxicity. This corroborates that enhancing Blm10/PA200 function can provide a promising potential therapeutic strategy to counteract αSyn‐induced cellular dysfunction.

### 3.3 *BLM10* Overexpression Promotes αSyn Clearance by Reducing Steady‐State Levels and Enhancing Its Turnover

**FIGURE 3:**
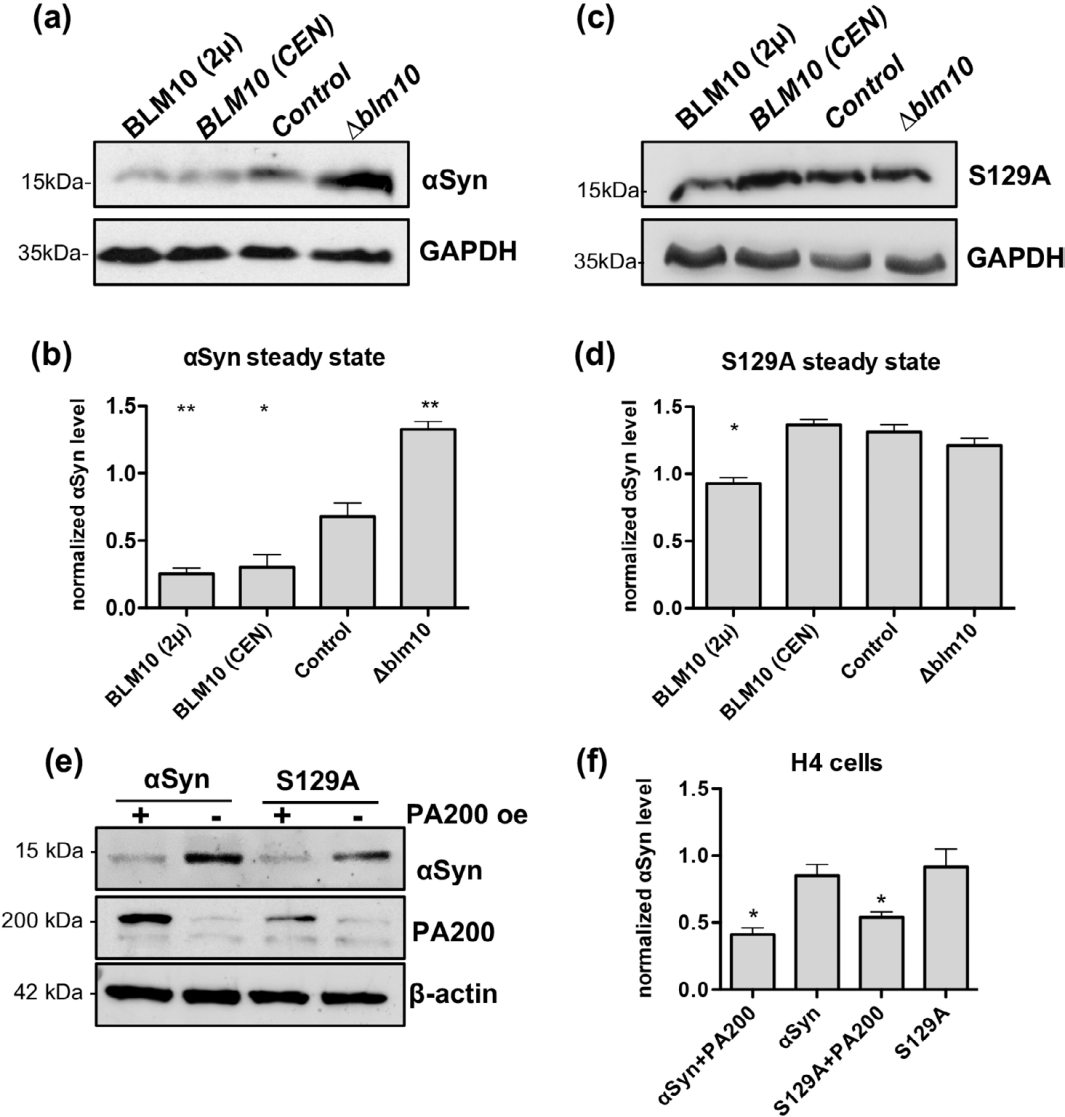
Elevated Blm10 levels reduce αSyn steady‐state levels. (a) Immunoblot of αSyn steady‐state levels after 6 h of *GAL1*‐driven expression in cells with varying *BLM10* expression levels. Control denotes endogenous *BLM10* expression in yeast cells with empty vector. (b) Quantification of αSyn levels from (a) normalized to GAPDH. Statistical significance was assessed by Student's *t*‐test relative to endogenous *BLM10* expression level (control) (**p < 0.01; *p < 0.05; *n* = 3). (c) Immunoblot of steady‐state levels of the phosphorylation‐deficient αSyn variant S129A after 6 h of *GAL1*‐driven induction under varying *BLM10* expression levels. (d) Quantification of S129A protein levels from (c) normalized to GAPDH. Statistical significance was evaluated by Student's *t*‐test relative to endogenous *BLM10* expression level (control) (*p < 0.05; *n* = 3). (e) Immunoblot of αSyn levels in H4 cells transfected with αSyn either alone or in combination with PA200. (f) Quantification of αSyn levels from (e) normalized to β‐Actin. Statistical significance was assessed by Student's *t*‐test (*p < 0.05; *n* = 3).

Previous studies have demonstrated that αSyn exerts a dose‐dependent cytotoxic effect in yeast, with higher cytosolic levels correlating with increased aggregate formation and enhanced toxicity (Petroi et al. 2012). Independently, Blm10‐CP assemblies have been shown to facilitate degradation of small, misfolded proteins such as Tau and Huntingtin (Aladdin et al. 2020; Dange et al. 2011). Steady‐state αSyn protein levels were analyzed using immuno‐hybridization to assess whether the protective effect of Blm10 in αSyn‐expressing cells is linked to a reduction of αSyn burden (Figure 3a,b). Overexpression of *BLM10* significantly reduced cellular αSyn levels, even at lower expression levels. A similar effect was observed in cells expressing the phosphorylation‐deficient S129A variant of αSyn, although the effect was attenuated (Figure 3c,d). Notably, a comparable reduction in αSyn steady‐state levels was observed in H4 cells upon overexpression of the human ortholog PA200, indicating that this effect is conserved across yeast and mammalian cells (Figure 3e,f).

**FIGURE 4:**
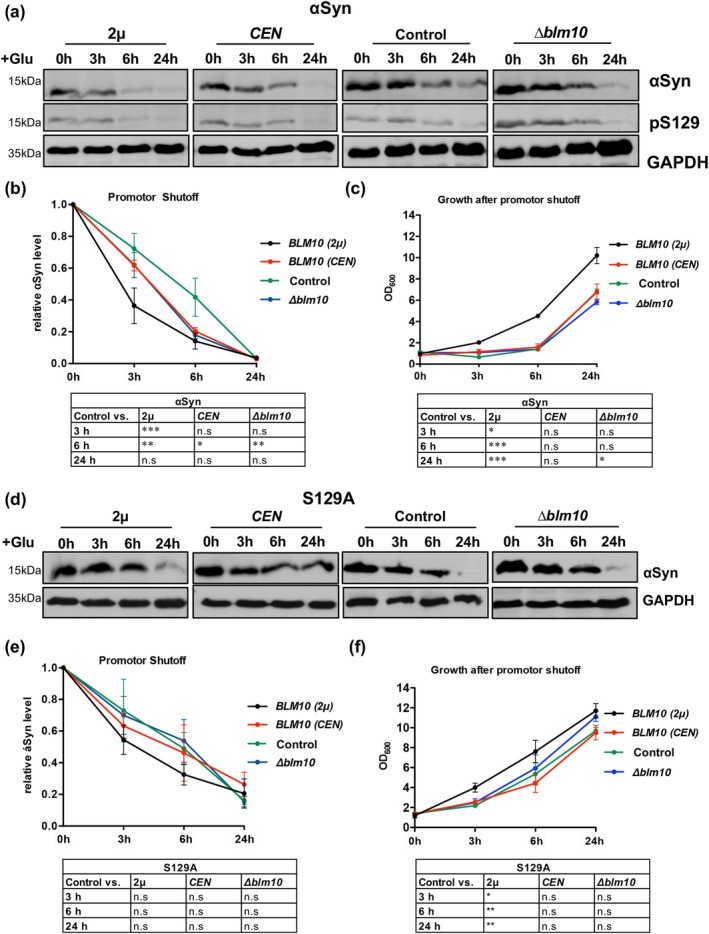
*BLM10* overexpression enhances αSyn turnover. (a) Immunoblot of αSyn protein levels after promoter shut‐off. expression of αSyn was induced for 6 h, then halted by addition of glucose. Samples were collected at indicated times post glucose addition (+Glu). Different *BLM10* expression levels were achieved using high‐copy (2 μ) and low‐copy (*CEN*) plasmids, which are compared against the endogenous expression levels of *BLM10* (Control) and *Δblm10*. The membranes were stripped and re‐probed with pS129‐specific antibody. (b) Quantification of αSyn levels from (a) relative to the levels prior to glucose addition (0 h). (c) Growth of yeast cells expressing *GAL1*‐driven αSyn in liquid culture after promoter shut‐off. (d) Immunoblot analysis of S129A protein levels after promoter shut‐off, comparing the same expression levels as described in (a). *GAL1* promoter‐driven expression was halted after 6 h by glucose addition, and samples were collected at indicated times. (e) Quantification of S129A protein levels from (d) normalized to levels before promoter shut‐off. (f) Yeast growth in liquid culture expressing *GAL1*‐driven S129A αSyn mutant after promoter shut‐off. Statistical analysis, shown beneath the corresponding graphs, was performed using a two‐way ANOVA with Dunnett's multiple comparison test comparing αSyn and its mutants to EV control (*p < 0.05; **p < 0.01; ***p < 0.001; n.s *p* > 0.05; *n* = 3).

Promoter shut‐off assays were performed to determine whether the reduction in αSyn levels was due to increased protein turnover. Expression of the αSyn‐encoding *SCNA* gene was driven by the yeast *GAL1* promoter, which is effectively repressed upon glucose addition. Following glucose‐induced transcriptional repression, αSyn degradation was monitored by immuno‐hybridization at indicated time points (Figure 4). Cells overexpressing *BLM10* exhibited markedly accelerated αSyn degradation compared to those expressing it at endogenous levels (Figure 4a,b), which correlated with enhanced cell growth during the shut‐off period (Figure 4c). This suggests that *BLM10* overexpression promotes αSyn clearance, thus mitigating cytotoxicity. The effect of *BLM10* overexpression was diminished in cells expressing the non‐phosphorylatable S129A variant (Figure 4d). Specifically, clearance rates did not significantly differ across *BLM10* expression levels, and cell growth remained largely unaffected by *BLM10* expression levels after promoter shut‐off (Figure 4e,f). These findings suggest that phosphorylation at serine 129 influences αSyn degradation by Blm10‐containing proteasome assemblies. Collectively, these results indicate that increased expression of *BLM10* reduces αSyn levels by promoting its degradation, potentially through enhanced assembly of Blm10‐CP proteasomes, which emerge as a prominent candidate for mediating proteasomal αSyn degradation.

### 3.4 Overexpression of *BLM10* / *PSME4* Rescues αSyn Induced Proteasome Inhibition In Vivo

**FIGURE 5:**
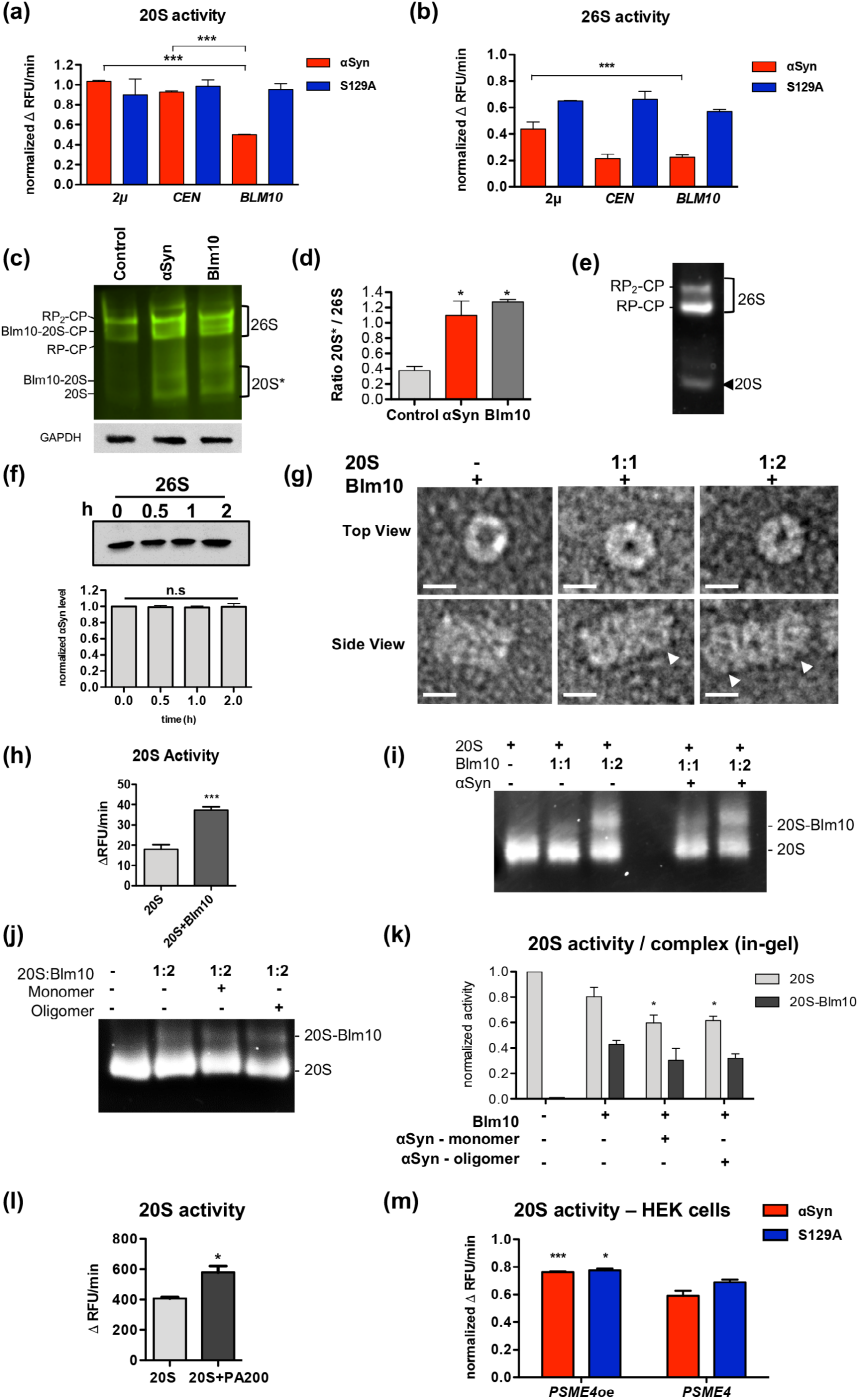
Blm10 rescues αSyn‐induced proteasome inhibition and promotes efficient proteasomal degradation. (a, b) 26S and 20S proteasome activity measured by SUC‐LLVY‐AMC assay in crude extracts from cells expressing αSyn or S129A with varying *BLM10* expression levels. Activity is presented as average change in relative fluorescence units (RFU) per minute and normalized to empty vector (EV) control for each *BLM10* condition. One‐way ANOVA with Dunnett's post hoc (***p < 0.001; *n* = 3). (c) Extracts from cells, expressing *PRE5*‐GFP with αSyn or *BLM10 (CEN)* were prepared in presence of ATP and resolved on 4% native PAGE. Fluorescence of Pre5‐GFP was imaged by a fluoroimager. Cells with empty vector were used as control. The protein bands assigned to the two isoforms of the 26S proteasome (RP_2_‐CP and RP‐CP), 20S, and Blm10‐capped 20S proteasomes, are indicated. (d) Relative fluorescence abundance of 20S and Blm10‐20S bands (indicated with 20S*) to 26S per lane, determined from GFP fluorescence from (c). One‐way ANOVA with Newman–Keuls multiple comparison test (*p < 0.05; *n* = 3) (e) In‐gel proteasome activity assay following native gel electrophoresis of 3 × FLAG‐purified 26S proteasomes. Proteasome activity was analyzed in the presence of ATP and 0.05% SDS, revealing single/double‐capped 26S and uncapped 20S. RP—regulatory particle. (f) In vitro degradation of αSyn by 26S proteasomes analyzed by immunoblot at indicated time points with αSyn antibody (upper panel). Quantification of αSyn levels at indicated times, normalized to time‐point 0 (n.s *p* > 0.05, *n* = 3) (lower panel). (f) Schematic representation of 20S reconstitution with Blm10. (g) Negative‐stain transmission electron microscopy of 20S proteasomes reconstituted with Blm10 at varying stoichiometric ratios. White arrows indicate Blm10 caps on 20S particles. Scale bar = 10 nm. (h) SUC‐LLVY‐AMC assay of purified 20S proteasomes with Blm10 at a 1:2 M ratio. Data represent the average RFU change per minute. Statistical significance was assessed by Student's *t*‐test (***p < 0.001; *n* = 3). (i) In‐gel SUC‐LLVY‐AMC activity assay following native gel electrophoresis, showing 20S proteasomes in complex with Blm10 in the absence or presence of 500 nM αSyn monomers. (j) In‐gel SUC‐LLVY‐AMC activity as in (i), comparing 20S reconstituted at a 1:2 ratio with Blm10 in the absence or presence of 500 nM αSyn monomers or oligomers. (k) Quantification of (j), showing change in RFU per minute normalized to 20S‐only control. For 20S samples, significance was calculated versus 20S alone; for 20S + Blm10 samples with αSyn, significance was calculated relative to 20S + Blm10 without αSyn. One‐way ANOVA with Dunnett's post hoc test was used (*p < 0.05; *n* = 3). (l) SUC‐LLVY‐AMC assay of purified 20S proteasomes with PA200 at a 1:2 M ratio. Data represent the average RFU change per minute. Statistical significance was assessed by Student's *t*‐test (*p < 0.05; *n* = 3). (m) 20S proteasome activity measured as in (a). Crude protein extracts were obtained from HEK cells with endogenous *PSME4* expression levels and cells with increased *PSME4* expression (*PSME4*oe). Statistical significance was determined by one‐way ANOVA with Dunnett's post hoc test (*p < 0.05;***p < 0.001; *n* = 3).

The interplay of αSyn expression and elevated Blm10 levels on the 26S or 20S proteasome assembly and resulting activities was examined. Crude protein extracts from yeast strains expressing different levels of *BLM10* were compared. The chymotrypsin‐like proteasome activity was measured by monitoring the degradation of the fluorogenic peptide SUC‐LLVY‐AMC in the presence or absence of ATP and Mg^2+^. This approach allowed us to distinguish between ATP‐dependent 26S and ATP‐independent 20S activities. Crude protein extracts treated with the proteasome inhibitor MG132 as a control revealed that the observed activity is specific for proteasomal cleavage (Figure S4). The contribution of Blm10 was assessed by measuring proteasome activity under ATP‐free conditions, where Blm10 specifically activates the latent 20S core particle by promoting gate opening. Assessment of 26S proteasome activity revealed a marked reduction in proteolytic function upon αSyn expression (Figure 5a). This inhibitory effect was less pronounced in cells expressing the S129A variant, indicating that phosphorylation at serine 129 contributes to αSyn‐mediated 26S inhibition. Overexpression of *BLM10* partially restored 26S proteasome activity in cells expressing αSyn. In contrast, *BLM10* expression had minimal impact on 26S activity in the presence of S129A, further indicating that the Blm10 rescue effect is dependent on the specific αSyn post‐translational modification. αSyn‐induced inhibition of 20S activity was less pronounced (Figure 5b). Importantly, overexpression of *BLM10*, even at moderate levels, robustly restored 20S activity in cells expressing αSyn. This effect was not observed in S129A‐expressing cells, where 20S activity remained largely unaffected irrespective of *BLM10* expression levels. Taken together, these results suggest that αSyn impairs proteasomal activity, particularly that of the 26S complex, in a pS129‐dependent manner. Whereas overexpression of *BLM10/PSME4* moderately alleviates 26S inhibition, it almost completely rescues the proteolytic impairment of the 20S proteasome.

To further assess whether αSyn expression influences proteasome composition, native PAGE analysis was performed using Pre5‐GFP‐tagged proteasomes. In control cells, predominantly single‐ (RP‐CP) and double‐capped (RP_2_‐CP) 26S proteasomes were detected, with minimal levels of free 20S core particles. In contrast, cells expressing αSyn or *BLM10* (*CEN*) exhibited a band corresponding to free 20S proteasomes, as well as a band consistent with Blm10‐capped 20S complexes, as confirmed by fluorescence imaging (Figure 5c,d). These observations indicate that αSyn expression, similar to *BLM10* overexpression, promotes a shift in proteasome composition toward free 20S and Blm10‐associated 20S assemblies, consistent with the observed stabilization of Blm10.

In vitro experiments were performed to compare the ability of 20S, 26S, and hybrid proteasomes to degrade αSyn. Intact 26S proteasomes were isolated from yeast cells by affinity purification using *RPN11‐3xFLAG* strain. The activities of the 30S double‐capped (RP_2_‐CP), 26S single‐capped (RP‐CP), or 20S proteasomes were visualized using Native PAGE followed by in‐gel activity assay (Figure 5e). The efficacy of 26S proteasomes to degrade recombinant αSyn in vitro was analyzed (Figure 5f). The degradation assay revealed that the 26S proteasome is unable to break down αSyn in the observed time frame in absence of additional co‐factors, in accordance with recent findings (Maestro‐López et al. 2026). To analyze whether Blm10‐capped 20S core particle is able to degrade unfolded αSyn, we reconstituted the core particle (CP) with Blm10 (Figure 5g). 20S and Blm10 protein were isolated from yeast by affinity purification using *PRE1‐3xFLAG* strain and *3xFLAG‐BLM10* expressing cells, respectively. 20S‐Blm10 proteasomes were reconstituted in vitro using 1:1 and 1:2 M ratios of 20S to Blm10. The complex formation was analyzed with negative‐stain transmission electron microscopy (TEM) (Figure 5g). Complex formation at molar ratio 1:1 resulted in predominantly single‐capped 20S‐Blm10 complexes and uncapped 20S particles, whereas at molar ratio 1:2 most of the complexes were double‐capped (Figure S5). Measurements of the enzymatic activity of the reconstituted complex validated that Blm10‐capped proteasomes have higher activities compared to uncapped 20S proteasomes (Figure 5h).

Native PAGE and in‐gel chymotrypsin activity assays also demonstrated successful reconstitution and activities of the 20S‐Blm10 proteasomes (Figure 5i). The presence of recombinant αSyn did not inhibit the activity of the 20S‐Blm10 complex. It was further analyzed whether αSyn oligomers might affect the assembly or activity of 20S‐Blm10 complexes. αSyn oligomers were purified using size exclusion chromatography (Figure S6a). The oligomeric fraction was analyzed with Coomassie staining and dot‐blot analysis using the oligomer‐specific A11 antibody (Figure S6b,c). These oligomers correspond to small, soluble species migrating at ~70 kDa on SDS‐PAGE, consistent with early, kinetically trapped intermediates that form under non‐agitating, low‐temperature conditions. The reconstitution mix was incubated with αSyn monomers or oligomers and subsequently loaded onto a native PAGE gel. SUC‐LLVY‐AMC hydrolysis shows that the activity of the 20S‐Blm10 complex remains unchanged in the presence of αSyn monomers or oligomers (Figure 5j,k). However, a reduction in 20S activity was visible in the αSyn‐containing samples.

The impact of αSyn monomers and oligomers on 20S proteasomes was further analyzed using SUC‐LLVY‐AMC hydrolysis assay in vitro, revealing a dose‐dependent inhibition of 20S proteasomes by αSyn monomers or oligomers (Figure S7a,b). The inhibition of activity could be partially rescued when 20S proteasomes were reconstituted with Blm10 (Figure S7c–e). Similarly, human 20S proteasomes were reconstituted with PA200, purified from yeast (Figure 5l). Reconstituted human proteasomes exhibited significantly higher activity compared to uncapped 20S proteasomes. PA200 reconstitution partially alleviated the inhibition induced by αSyn monomers (Figure S7f). The rescue effect was less pronounced when PA200‐20S proteasomes were incubated with αSyn oligomers (Figure S7g,h). Similar results were obtained when analyzing 20S activity in HEK cell crude protein extracts (Figure 5m). Expression of αSyn or the phosphodeficient S129A variant significantly reduces 20S activity, which can be partially rescued by overexpression of *PSME4*. These results reveal that αSyn affects the activity of the 20S proteasome in its uncapped form, whereas Blm10‐capped 20S complexes retain their proteolytic function even in the presence of αSyn monomers and oligomers, highlighting Blm10/PA200's protective role against αSyn‐induced proteasomal impairment.

### 3.5 Blm10/PA200 Capping Facilitates αSyn Clearance

**FIGURE 6:**
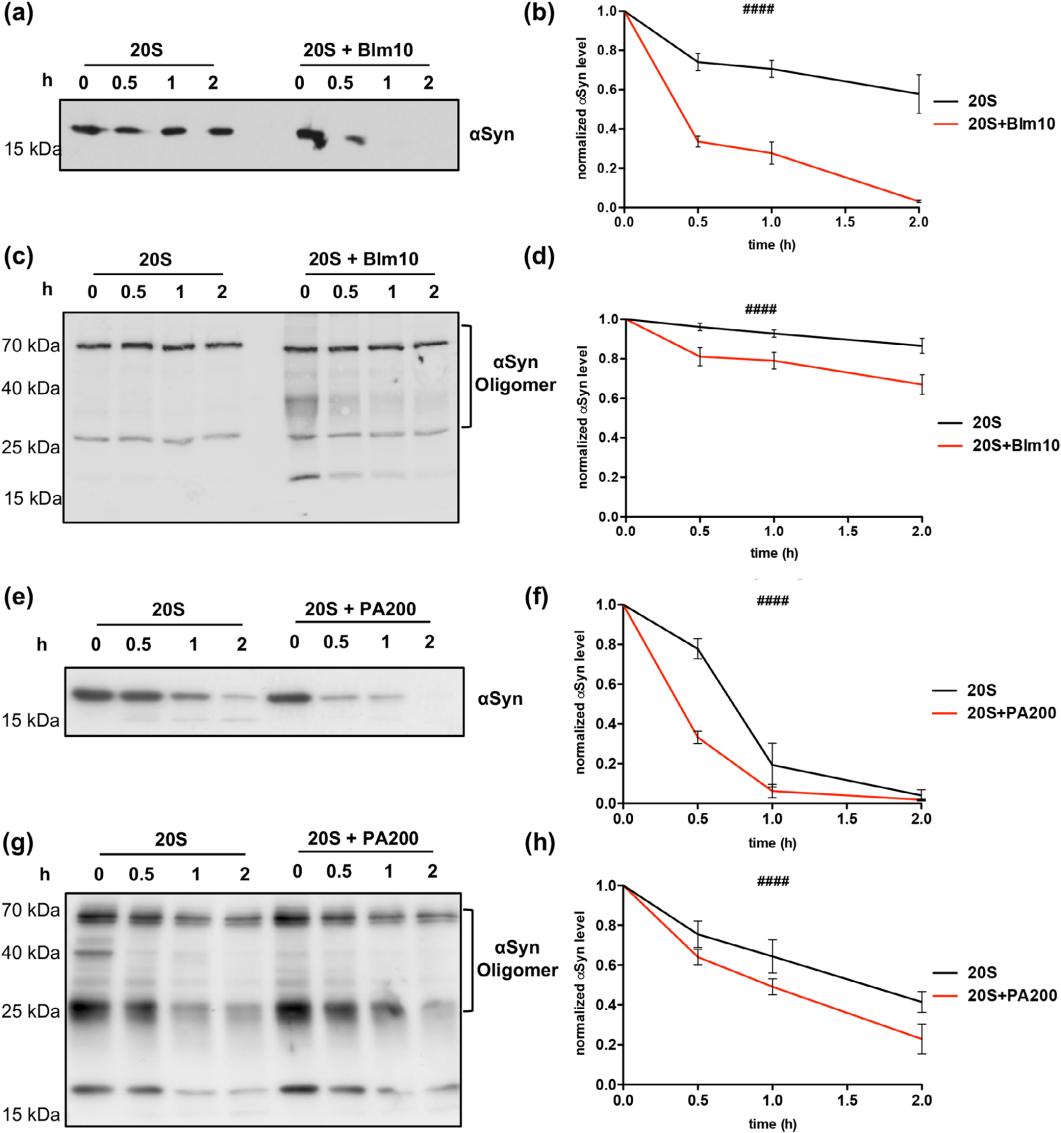
20S‐Blm10 proteasome efficiently degrades αSyn in vitro. (a) In vitro degradation of αSyn monomers by yeast 20S or Blm10‐reconstituted 20S proteasomes. Reactions contained 2.5 μg of purified 20S and, where indicated, two‐fold molar excess of Blm10, with 0.5 μg αSyn monomers at the reaction start. Degradation was monitored over time by immunoblot using αSyn antibody. (b) Quantification of αSyn degradation from (a), normalized to time‐point 0. (c) In vitro degradation of αSyn oligomers by yeast 20S or Blm10‐reconstituted 20S proteasomes. Reactions contained 2.5 μg of purified 20S and, where indicated, two‐fold molar excess of Blm10, with 0.5 μg αSyn oligomers at the reaction start. Degradation was monitored over time by immunoblot using αSyn antibody. (d) Densitometric quantification of αSyn oligomer degradation from (c), normalized to time‐point 0. (e) In vitro degradation of αSyn monomers by human 20S or PA200‐reconstituted 20S proteasome. Reactions contained 1 μg commercially purified 20S and, where indicated, two‐fold molar excess of PA200 with 0.5 μg αSyn monomers at the reaction start. Degradation was monitored over time by immunoblot using αSyn antibody. (f) Quantification of αSyn degradation from (e), normalized to time‐point 0. (g) In vitro degradation of αSyn oligomers by human 20S or PA200‐reconstituted 20S proteasome. Reactions contained 1 μg commercially purified 20S and, where indicated, two‐fold molar excess of PA200 with 0.5 μg αSyn oligomers at the reaction start. Degradation was monitored over time by immunoblot using αSyn antibody. (h) Quantification of αSyn degradation from (g), normalized to time‐point 0. All quantifications were assessed for statistical significance by ordinary two‐way ANOVA (*####p* < 0.0001; *n* = 3).

Purified recombinant αSyn was incubated with either 20S proteasomes or Blm10 reconstituted 20S proteasomes (20S‐Blm10 complexes) to determine whether the previously observed rescue effect of Blm10 on 20S activity is due to enhanced αSyn degradation. The efficiency of αSyn degradation was analyzed by immuno‐hybridization (Figure 6a), and quantified over time (Figure 6b). The 20S proteasomes displayed only limited ability to degrade αSyn, although their efficacy exceeded that of the 26S proteasomes. In contrast, Blm10‐capped 20S complexes exhibited markedly enhanced proteolytic activity, removing approximately 70% of αSyn within 1 h. These findings demonstrate that Blm10 significantly enhances the proteolytic activity of the 20S proteasome to degrade monomeric αSyn.

Given that A11‐positive oligomers have been identified as major contributors to αSyn mediated cytotoxicity and proteasome inhibition (Thibaudeau et al. 2018; Winner et al. 2011), we assessed the ability of Blm10‐capped proteasomes to degrade these oligomers (Figure 6c,d). αSyn forms oligomer species ranging from dimers/trimers to larger assemblies exceeding several hundred kDa, depending on preparation conditions (Chen and Cremades 2018). The small, soluble oligomers used in this study represent early intermediates within the broader spectrum of αSyn assemblies that coexist with higher‐molecular‐weight complexes (Figure S8a–c). These early oligomeric species likely retain structural flexibility and partially disordered regions, which may facilitate their engagement with the 20S proteasome.

Blm10‐capped 20S proteasomes moderately degraded αSyn oligomers in vitro into smaller assemblies. Degradation products of approximately 40 kDa and 25 kDa appeared at the earliest time‐point and were partially degraded over the 2‐h incubation. In contrast, uncapped 20S proteasomes neither reduced oligomer levels nor generated detectable degradation products.

To validate the findings with Blm10‐capped 20S proteasomes, PA200 was reconstituted with human 20S proteasomes, and αSyn degradation was assessed similarly. PA200‐capped 20S proteasomes degraded αSyn monomers significantly more efficiently in vitro, although the difference between capped and uncapped proteasomes was less pronounced than in yeast proteasomes (Figure 6e,f). Moreover, the degradation of αSyn oligomers was markedly enhanced in PA200‐capped proteasomes compared to uncapped ones (Figure 6g,h). These results suggest that the increased turnover efficiency is conserved between Blm10‐capped yeast proteasomes and PA200‐capped human proteasomes.

## 4 Discussion

αSyn accumulation perturbs cellular proteostasis and contributes to the pathogenesis of Parkinson's disease. Although intrinsically disordered proteins such as αSyn can be degraded by the 20S proteasome, the regulatory mechanisms that sustain this pathway under proteotoxic stress remain incompletely understood, particularly when canonical ubiquitin‐dependent degradation is compromised. We demonstrate that αSyn‐induced inhibition of autophagy stabilizes the proteasome activator Blm10. While PA200/Blm10 has previously been described as a proteasome activator, our study identifies a previously unrecognized proteostasis adaptation mechanism triggered by αSyn stress. These specialized proteasome complexes efficiently degrade both monomeric and oligomeric αSyn and remain resistant to αSyn‐mediated proteasome inhibition. Our findings therefore reveal a stress‐responsive proteasome configuration that maintains proteolysis under conditions where canonical 26S proteasomes are impaired, providing a new mechanistic link between autophagy inhibition, proteasome regulation, and αSyn proteostasis.

The pathological aggregation of αSyn into amyloid fibrils is a central mechanism underlying dopaminergic neuron degeneration in PD. This aggregation process is closely linked to the collapse of proteostasis, the dynamic regulation of protein synthesis, folding, and degradation (Lehtonen et al. 2019). Two key protein degradation systems contribute to cellular proteostasis: the ubiquitin‐proteasome system (UPS) and autophagy. Both macroautophagy and chaperone‐mediated autophagy are known to facilitate αSyn turnover (Vogiatzi et al. 2008; Webb et al. 2003), yet these processes are dysfunctional in PD patients, with αSyn itself acting as one of the contributors to this inhibition (Fellner et al. 2021). In addition to compromised autophagy, αSyn negatively impacts UPS function. We previously reported that αSyn stabilizes the proteasome assembly chaperone Rpn14, leading to reduced formation of fully active 26S proteasomes (Galka et al. 2024), accompanied by a reduced abundance of proteasomal subunits (Popova, Galka, et al. 2021).

In the current study, we extend these findings by exploring the impact of αSyn on the 20S proteasome and its regulator Blm10. Our results demonstrate that αSyn inhibits the 26S proteasome more severely than the 20S core particle. This is particularly significant as the 20S core particle is catalytically active even in the absence of ATP, unlike the 26S proteasome, which depends on ATP for substrate unfolding and deubiquitination via the 19S regulatory particle (Bard et al. 2018). The activity of the 20S proteasome can be regulated by modulators different than the 19S regulatory particle. This positions the 20S proteasome as a promising alternative degradation route for αSyn. Blm10, the yeast ortholog of human PA200, loosely binds to the 20S α‐ring. Both orthologues possess a conserved C‐terminal HbYX motif, capable of inducing partial gate opening and enhancing proteolysis (Dange et al. 2011). Prior studies have demonstrated that Blm10‐ and PA200‐capped 20S proteasomes efficiently degrade misfolded disease‐associated proteins such as tau and huntingtin (Aladdin et al. 2020; Dange et al. 2011). Here, we demonstrate comprehensively that the Blm10 and PA200‐capped proteasomes are key cellular antagonists against the aggregation of amyloid proteins, using the PD related protein αSyn as an example. Our findings therefore suggest that cells may adapt proteasome function under conditions of αSyn‐induced proteotoxic stress by shifting toward alternative proteasome configurations that do not rely on canonical ubiquitin‐dependent degradation.

This work highlights Blm10‐CP as an efficient turnover machine for monomeric αSyn, which is also capable of degrading A11‐positive oligomers of αSyn, a thus far unknown function. The performed experiments highlight that the in vitro observations translate into an in vivo context, through the use of cellular PD models. In vivo, *BLM10* overexpression enhances αSyn turnover, reduces aggregation, and restores 20S activity, improving cell viability. Overexpression of *PSME4* also significantly reduces the amount of αSyn inclusions within H4 cells and reduces the steady state levels of αSyn in vivo. These novel findings identify 20S proteasomes capped with Blm10 or PA200 as effective αSyn degradation units. Importantly, our results indicate that Blm10/PA200 does not act through direct physical interaction with αSyn but rather through activation of the 20S proteasome, thereby facilitating degradation of intrinsically disordered αSyn species in a ubiquitin‐independent manner. This supports a model in which Blm10/PA200 enhances the proteolytic capacity of the 20S proteasome rather than acting as a substrate‐specific adaptor that directly recruits αSyn. Our data further suggest that *BLM10* overexpression partially rescues 26S proteasome activity, potentially through the formation of hybrid proteasomes, in which the 20S core is capped by the 19S regulatory particle on one side and Blm10 on the other. Although the function of such hybrid proteasomes remains incompletely understood, previous findings (Burris et al. 2021) indicate that Blm10 overexpression alters proteasome composition in favor of non‐19S capped complexes. Increased formation of Blm10‐capped proteasomes may therefore represent a stress‐adaptive mechanism that preserves protein degradation capacity when canonical 26S proteasome function becomes compromised.

The physiological relevance of this mechanism is highlighted by the conserved function of PA200 in humans. Despite limited sequence similarity, both Blm10 and PA200 function analogously by binding the 20S α‐ring and promoting gate opening mediated by the HbYX motif (Yazgili et al. 2022). Importantly, expression of both *BLM10* and *PSME4* declines with age (Chen et al. 2020), potentially contributing to the impaired proteasomal clearance of aggregation prone proteins in age‐related diseases such as PD. Moreover, *PSME4* expression is significantly reduced in the blood of PD patients compared to age‐matched controls (Yuan et al. 2020), which points to a vulnerability in this proteasome assembly during aging and neurodegeneration. Diminished Blm10/PA200 levels likely impair clearance of αSyn under pathological conditions. PA200 is also involved in the turnover of acetylated histones, a function shared with Blm10 and linked to nuclear proteostasis (Chen et al. 2020). Since αSyn localizes to the nucleus and interferes with histone acetylation (Kontopoulos et al. 2006), nucleosomes may represent a critical site of interaction between αSyn and Blm10/PA200‐capped proteasomes. We observed increased Blm10 protein stability upon αSyn expression. However, this stabilization is unlikely to fully compensate for reduced transcriptional levels. Blm10, like PA200, is tightly regulated and present at sub‐stoichiometric levels to proteasomes in the cell (Burris et al. 2021). Alternatively, it is possible that the increased stability of Blm10/PA200 could suppress transcription via potential autoregulation. Future studies in neuronal systems will be required to determine how PA200‐mediated 20S proteasome activation contributes to αSyn turnover in dopaminergic neurons and to evaluate its potential relevance for PD pathology.

We show a mechanistic link between αSyn‐induced autophagy inhibition and Blm10 stabilization. Blm10 is predominantly degraded through autophagy (Burris et al. 2021), whereby αSyn inhibits autophagic flux, specifically autophagosome‐vacuole fusion, which has been previously observed in mammalian cells with αSyn pathology (Tang et al. 2021). Impairment of autophagic flux leads to Blm10 stabilization. Notably, this stabilization depends on αSyn phosphorylation at serine 129, further implicating this modification in proteostasis regulation. This reveals a connection between αSyn‐induced autophagy impairment and proteasome remodeling. By stabilizing Blm10, inhibition of autophagic flux indirectly promotes the formation of Blm10‐capped 20S proteasomes, thereby linking two major proteostasis pathways in a coordinated cellular response to αSyn stress.

Our study provides novel mechanistic insights into αSyn‐induced proteasome inhibition. In vitro assays revealed that both αSyn oligomers and monomers inhibit 20S activities. This supports prior findings (Thibaudeau et al. 2018) that A11‐positive oligomers are potent 20S inhibitors that stabilize a closed proteasome conformation, blocking activation by HbYX‐containing regulators. Our observation that monomers also inhibit the 20S core considerably expands this paradigm and suggests a much broader toxic potential of αSyn species. Importantly, Blm10 binding restores activities of the 20S proteasome particles in the presence of both monomeric and oligomeric αSyn, indicating its protective role. Moreover, this effect is conserved when human 20S proteasomes are reconstituted with PA200. Importantly, the observed in vitro effect can be recapitulated by overexpression of *BLM10* or *PSME4*. Together, these findings support the role of Blm10/PA200‐capped 20S proteasomes as a specialized proteasome configuration capable of maintaining proteolytic activity under conditions of αSyn‐induced proteotoxic stress.

In conclusion, our findings demonstrate that αSyn monomers and oligomers inhibit 20S proteasome function. Most likely this is achieved by stabilizing a closed‐gate conformation of the 20S proteasome. Blm10/PA200 binding counteracts this effect, which results in restored proteolytic activity, promoting αSyn clearance. This highlights a unique role for Blm10/PA200‐capped 20S proteasomes in maintaining proteostasis under proteotoxic stress. The results provide a new promising perspective, which points to novel therapeutics with potential uses against neurodegenerative diseases including PD as well as other aggregopathies. Our study therefore uncovers a stress‐adaptive proteasome remodeling mechanism in which stabilization of Blm10/PA200 promotes the formation of activated 20S proteasomes that efficiently degrade αSyn species.

## Author Contributions

Conceptualization: T.T.A., B.P., G.H.B., T.F.O., and E.S. Funding acquisition: G.H.B. Investigation: T.T.A., A.Z., M.M., Z.B., and B.P. Supervision: B.P., T.F.O., and G.H.B. Writing – original draft: T.T.A., B.P., and G.H.B. Writing – review and editing: T.T.A., B.P., E.S., T.F.O., and G.H.B.

## Funding

This work was supported by the Deutsche Forschungsgemeinschaft (DFG BR1502/21‐1 to GB). T.F.O. was supported by DFG SFB1286, Project B8. This work was partly supported by the Göttingen Graduate Centre for Neurosciences, Biophysics and Molecular Biosciences at the Georg‐August Universität Göttingen. M.M. is supported by DAAD Research Grants—Doctoral Programmes in Germany (57552340).

## Conflicts of Interest

The authors declare no conflicts of interest.

## Supporting information


**Table S1:** Yeast strains used in this study.
**Table S2:** Plasmids used in this study.
**Table S3:** Antibodies used in this study.

**Figure S1:** Hyperphosphorylation of αSyn stabilizes Blm10 similarly to the phospho‐mimicking mutant S129D.
**Figure S2:** Blm10 and αSyn do not interact physically.
**Figure S3:** αSyn inhibits autophagy.
**Figure S4:** Reconstitution of 20S proteasomes with Blm10.
**Figure S5:** Proteasome activity assays with crude protein extracts.
**Figure S6:** Purification of αSyn oligomers for activity assays.
**Figure S7:** αSyn oligomers and monomers inhibit 20S proteasome.
**Figure S8:** Characterization of oligomeric species.

## Data Availability

The data that support the findings of this study are available from the corresponding author upon reasonable request.
