# Supplementary material for: Blm10/PA200‐Activated 20S Proteasomes Promote α‐Synuclein Degradation and Bypass Proteasome Inhibition in Parkinson's Disease Models
Source: Aging Cell. 2026 May 28;25(6):e70566. doi: 10.1111/acel.70566 (PMC13240070; doi:10.1111/acel.70566)
Supplement: Supplementary file 1 — Table S1: Yeast strains used in this study. Table S2: Plasmids used in this study. Table S3: Antibodies used in this study. [file ACEL-25-e70566-s001.pdf]

Supplementary Information

Supplementary Tables

Table S1. Yeast strains used in this study.

Name	Description	Source
W303-1A	MAT*a, ura3–1, trp1–1, leu2–3_112, his3–11, ade2–1, can1–100*	EUROSCARF
BY4741	MAT*a, ura3Δ0, his3Δ1, leu2Δ0, met15Δ0*	EUROSCARF
BLM10-tFT	YMaM330 *BLM10::mCherry::sfGFP*	(Khmelinskii et al., 2012)
BLM10-tFT Δ*atg7*	YMaM330 *BLM10-tFT*, *Δatg7::KanMX4*	this study
PRE1-3xFLAG	W303 *ade2-1, ura3-1, his3-11,25, trp1-1, leu2-3,112, can1, pre1::PRE1-3xFLAG-HIS3*	(Saeki et al., 2009)
PRE5-GFP	*BY4741 MATa; his3Δ1, leu2Δ0; met15Δ0; ura3Δ0 , pre5::PRE5-GFP-HIS3*	Invitrogen yeast GFP collection
RPN11-3xFLAG	W303 *ade2-1, ura3-1, his3-11,15, trp1-1, leu2-3,112, can1, rpn11::RPN11-3xFLAG*	(Saeki et al., 2009)
BY4741Δ*blm10*	BY4741 *MATa; his3Δ1; leu2Δ0; met15Δ0; ura3Δ0 Δblm10:NatMX4*	Invitrogen deletion collection
RH3468	MAT*α*; *ura3-52; trp1D2; leu2-3_112; his3-11; ade2-1; can1-100 GAL1::SNCA::GFP::URA3* (3 copies)	(Petroi et al., 2012)
EGY48	MATa, *his3, trp1, ura3, LexAop (x6)-LEU2*	(Golemis et al., 1999)

Table S2. Plasmids used in this study.

Name	Description	Source
p426	*2µ, URA3, $GAL1_{pr}$, $CYC1_{term}$, AmpR*	(Mumberg et al., 1994)
pME3760	p426-*$GAL1^{pr}$::SNCA*	(Petroi et al., 2012)
pME3763	p426-*$GAL1^{pr}$::SNCA-GFP*	(Petroi et al., 2012)
pME5320	p426-*$GAL1^{pr}$::$SNCA^{S129A}$*	(Popova et al., 2021a)
pME5570	p426-*$GAL1_{pr}$::$SNCA^{S129D}$*	this study
p425	*2µ, LEU2, GPD_{pr}, $CYC1_{term}$, AmpR*	(Mumberg et al., 1994)
pME5571	*CEN, LEU2, GPD_{pr}, $CYC1_{term}$, AmpR, mCherryBlm10*	this study
pME5572	p425-*mCherry-BLM10*	this study
pME5574	p426-*3xFLAG-BLM10*	this study
pME5575	p426-*3xFLAG-PA200*	this study
pRS315-EGFP-Atg8	*CEN, LEU2, $CYC1_{term}$, EGFP-ATG8*	(Voigt and Pöggeler, 2013)
pME4913	pET22b-*SNCA*	(Popova et al., 2021c)
pEG202	*2µ, HIS3, ADH^{pr}, LexA, ADH^{term}*	(Golemis et al., 1999)
pJG4-5	*2µ, TRP1, $GAL1^{pr}$, B42, ADH^{term}*	(Golemis et al., 1999)
pME5525	pEG202-*$SNCA^{WT}$*	(Galka et al., 2024)
pME5530	pEG202-*$SNCA^{S129A}$*	(Galka et al., 2024)
pME5573	pJG4-5-*BLM10*	this study
pME5090	p426-*$GAL1^{pr}$::VenusN*	(Popova, et al. 2021)
pME5036	p423-*$GAL1^{pr}$::VenusC*	(Popova, et al. 2021)
pME5033	p423-*GAL1::SNCA::VenusC*	(Tenreiro et al., 2016)
pME5034	p426-*GAL1::VenusN::SNCA*	(Tenreiro et al., 2016)
pME5517	p423-*GAL1pr::SNCAS129A::VenusC*	(Galka et al., 2024)
pME5518	p423-*GAL1pr::SNCAS129D::VenusC*	(Galka et al., 2024)
pME5577	p423-*GAL1::BLM10::VenusC*	this study
pME5578	pcDNA 3.1 with 6xHis-PA200	this study

Table S3. Antibodies used in this study.

Name	concentration	Origin	Source
α-RFP	1:5000	rat	Proteintech Group, Rosemont, IL
α-GFP	1:1000	rat	Proteintech Group, Rosemont, IL
α-FLAG	1:5000	mouse	Proteintech Group, Rosemont, IL
α-αSyn	1:2000	mouse	Becton Dickinson, Franklin Lakes, NJ
α-αSyn pS129	1:2000	mouse	WAKO chemicals, Richmond, VA
α-A11	1:1000	rabbit	Thermo Fischer, Waltham, MA
α-GAPDH	1:5000	mouse	Thermo Fischer, Waltham, MA
α-rat-HRP	1:1000	goat	Thermo Fischer, Waltham, MA
α-mouse-HRP	1:5000	goat	Thermo Fischer, Waltham, MA
α-rabbit-HRP	1:5000	goat	Thermo Fischer, Waltham, MA
α-PA200	1:1000	rabbit	Thermo Fischer, Waltham, MA
α-β-Actin	1:10000	mouse	Thermo Fischer, Waltham, MA

Supplementary Methods

Yeast-Two Hybrid assay

Putative protein-protein interaction were analyzed using yeast-two-hybrid assay as previously described (Golemis et al., 1999). Proteins are genetically fused to either a DNA binding domain or an activation domain of a transcriptional activator. α-synuclein and its variants S129A and S129D were fused to the activation domain, while Blm10 was attached to the DNA binding domain of the bacterial repressor protein LexA. Constructs were co-transformed into the yeast strain EGY48 and interaction was analyzed using selective SC-His-Trp-Leu plates supplemented with 2% galactose or 2% glucose as a negative control.

Bimolecular fluorescence complementation assay

Potential interactions between Blm10 and αSyn or its variants were analyzed using bimolecular fluorescence complementation (BiFC) assay (Hu et al., 2002). The Venus

fluorophore was split into C- and N-terminal fragments, which were genetically fused to the proteins of interest. Blm10 was fused with the N-terminal fragment, whereas αSyn, S129A, and S129D were fused to the C-terminal fragment. The constructs were co-transformed into the BY4741 yeast strain and expressed, followed by fluorescence microscopy. Protein-protein interaction was indicated by a reconstituted Venus signal detectable in the GFP channel.

Nitrogen Starvation

Transformed yeast cells were grown overnight in selective SC medium supplemented with 2% raffinose. The cultures were then diluted to an OD_{600} of 0.3 and re-inoculated in SC medium containing 2% galactose to induce *GAL1*-driven expression of αSyn and its mutants for 6 h. After induction, samples were collected for microscopic analysis and protein extraction. The remaining cells were washed twice and transferred to nitrogen-deficient SC medium containing 0.2% galactose. After 16 h of starvation, cells were harvested for protein extraction and microscopic analysis.

References

Galka, D., Ali, T. T., Bast, A., Niederleithinger, M., Gerhardt, E., Motosugi, R., et al. (2024). Inhibition of 26S proteasome activity by α-synuclein is mediated by the proteasomal chaperone Rpn14/PAAF1. *Aging Cell* 23. doi:10.1111/acel.14128.

Golemis, E. A., Serebriiskii, I., and Law, S. F. (1999). The yeast two-hybrid system: criteria for detecting physiologically significant protein-protein interactions. *Curr. Issues Mol. Biol.* 1, 31–45. doi:10.21775/cimb.001.031.

Hu, C. D., Chinenov, Y., and Kerppola, T. K. (2002). Visualization of Interactions among bZIP and Rel Family Proteins in Living Cells Using Bimolecular Fluorescence Complementation. *Mol. Cell* 9, 789–798. doi:10.1016/S1097-2765(02)00496-3.

Khmelinskii, A., Keller, P. J., Bartosik, A., Meurer, M., Barry, J. D., Mardin, B. R., et al. (2012). Tandem fluorescent protein timers for in vivo analysis of protein dynamics. *Nat. Biotechnol.* 30, 708–714. doi:10.1038/nbt.2281.

Mumberg, D., Muller, R., Funk, M., Müller, R., and Funk, M. (1994). Regulatable

promoters of Saccharomyces cerevisiae: comparison of transcriptional activity and their use for heterologous expression. *Nucleic Acids Res.* 22, 5767–8.

Petroi, D., Popova, B., Taheri-Talesh, N., Irniger, S., Shahpasandzadeh, H., Zweckstetter, M., et al. (2012). Aggregate clearance of alpha-synuclein in *Saccharomyces cerevisiae* depends more on autophagosome and vacuole function than on the proteasome. *J Biol Chem* 287, 27567–27579. doi:M112.361865 [pii]10.1074/jbc.M112.361865.

Popova, B., Galka, D., Häffner, N., Wang, D., Schmitt, K., Valerius, O., et al. (2021a). α-Synuclein Decreases the Abundance of Proteasome Subunits and Alters Ubiquitin Conjugates in Yeast. *Cells 2021, Vol. 10, Page 2229* 10, 2229. doi:10.3390/CELLS10092229.

Popova, B., Wang, D., Pätz, C., Akkermann, D., Lázaro, D. F., Galka, D., et al. (2021b). DEAD-box RNA helicase Dbp4/DDX10 is an enhancer of α-synuclein toxicity and oligomerization. *PLOS Genet.* 17, e1009407. doi:10.1371/journal.pgen.1009407.

Popova, B., Wang, D., Rajavel, A., Dhamotharan, K., Lázaro, D. F., Gerke, J., et al. (2021c). Identification of Two Novel Peptides That Inhibit α-Synuclein Toxicity and Aggregation. *Front. Mol. Neurosci.* 14, 54. doi:10.3389/FNMOL.2021.659926.

Saeki, Y., Toh-e, A., Kudo, T., Kawamura, H., and Tanaka, K. (2009). Multiple Proteasome-Interacting Proteins Assist the Assembly of the Yeast 19S Regulatory Particle. *Cell* 137, 900–913. doi:10.1016/J.CELL.2009.05.005.

Tenreiro, S., Rosado-Ramos, R., Gerhardt, E., Favretto, F., Magalhães, F., Popova, B., et al. (2016). Yeast reveals similar molecular mechanisms underlying alpha- and beta-synuclein toxicity. *Hum. Mol. Genet.* 25, 275–290. doi:10.1093/hmg/ddv470.

Voigt, O., and Pöggeler, S. (2013). Autophagy genes Smatg8 and Smatg4 are required for fruiting-body development, vegetative growth and ascospore germination in the filamentous ascomycete Sordaria macrospora. *Autophagy* 9, 33–49. doi:10.4161/AUTO.22398.
