# Supplementary material for: Blm10/PA200‐Activated 20S Proteasomes Promote α‐Synuclein Degradation and Bypass Proteasome Inhibition in Parkinson's Disease Models
Source: Aging Cell. 2026 May 28;25(6):e70566. doi: 10.1111/acel.70566 (PMC13240070; doi:10.1111/acel.70566)
Supplement: Supplementary file 2 — Figure S1: Hyperphosphorylation of αSyn stabilizes Blm10 similarly to the phospho‐mimicking mutant S129D. Figure S2: Blm10 and αSyn do not interact physically. Figure S3: αSyn inhibits autophagy. Figure S4: Reconstitution of 20S proteasomes with Blm10. Figure S5: Proteasome activity assays with crude protein extracts. Figure S6: Purification of αSyn oligomers for activity assays. Figure S7: αSyn oligomers and monomers inhibit 20S proteasome. Figure S8: Characterization of oligomeric species. [file ACEL-25-e70566-s002.pdf]

Supplementary Figures

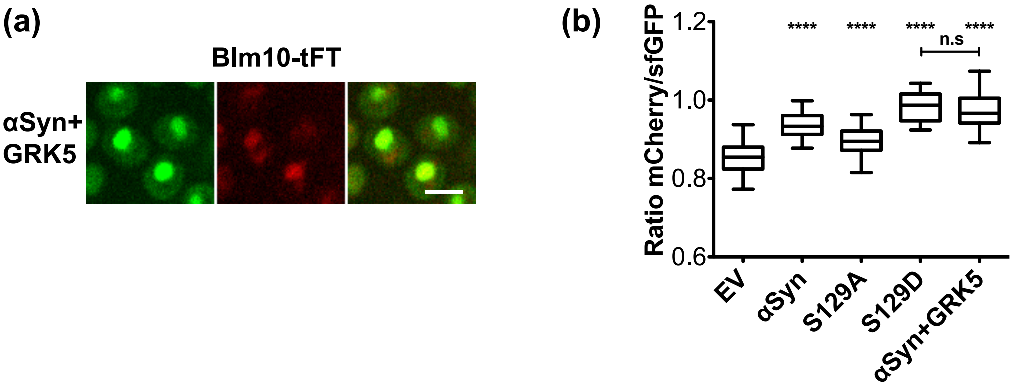

Figure S1. Hyperphosphorylation of αSyn stabilizes Blm10 similarly to the phospho-mimicking mutant S129D. (a) Tandem Fluorescent Timer (tFT) analysis of Blm10 stability by fluorescence microscopy. Cells express *GAL1*-driven αSyn and constitutively expressed *GRK5*. Images were acquired after 6 h of αSyn induction. Scale bar = 5 µm. **(b)** Quantification of the mCherry to sfGFP fluorescence ratio from microscopy images, compared to samples shown in Figure 1. Fluorescence ratios were calculated for single cells. Statistical significance was determined by one-way ANOVA with Dunnett's post hoc test (****$p < 0.0001$; n.s $p > 0.05$; n = 50) in comparison to control empty vector (EV).

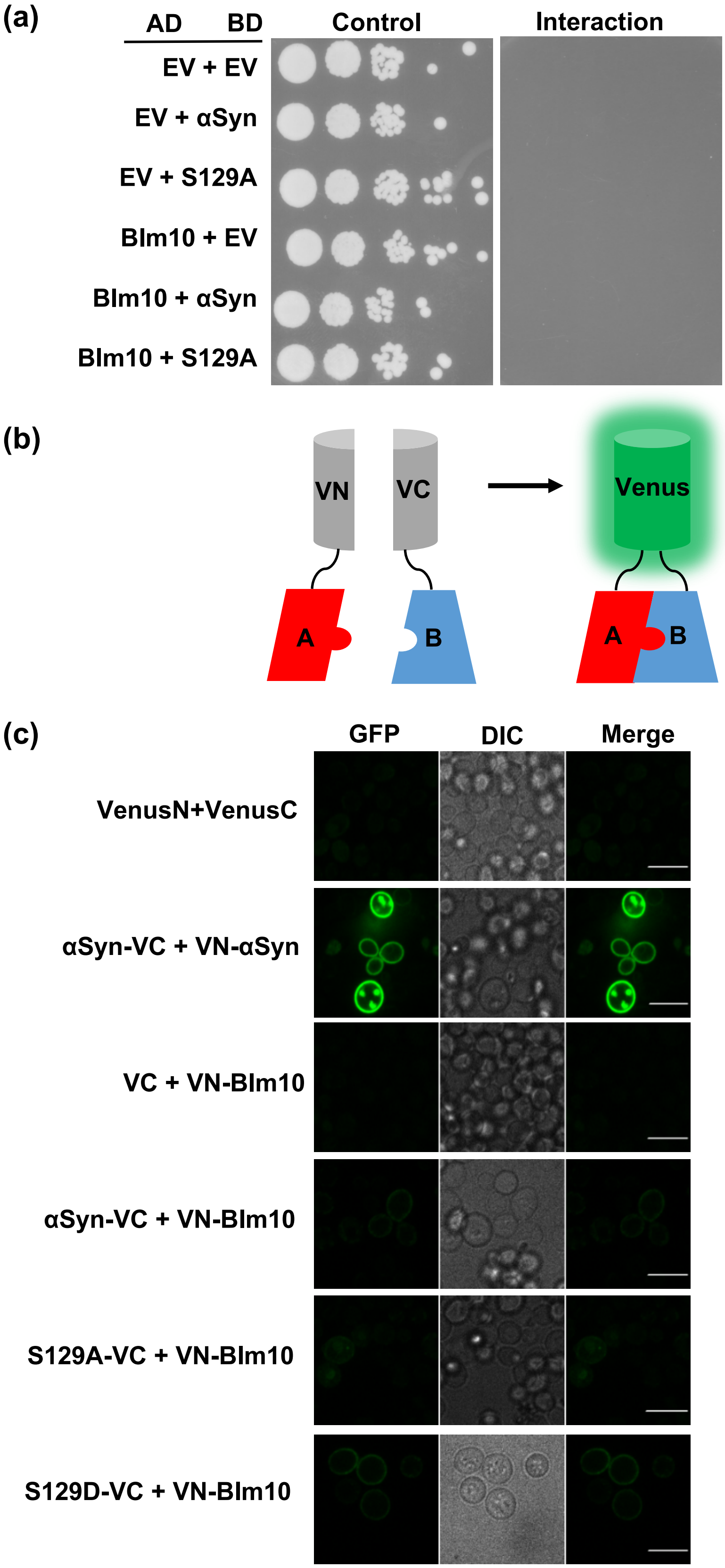
(a)
AD BD
Control
Interaction
EV + EV
EV + αSyn
EV + S129A
Blm10 + EV
Blm10 + αSyn
Blm10 + S129A
(b)
VN
VC
Venus
A
B
(c)
GFP
DIC
Merge
VenusN+VenusC
αSyn-VC + VN-αSyn
VC + VN-Blm10
αSyn-VC + VN-Blm10
S129A-VC + VN-Blm10
S129D-VC + VN-Blm10

Figure S2. Blm10 and αSyn do not interact physically. Yeast-two-hybrid assay using yeast cells transformed with plasmids encoding proteins fused to either a transcriptional activation domain (AD) or a DNA-binding domain (BD). The B42 activation domain-fused protein (prey) is under *GAL1* promotor control, while the LexA-DNA BD (bait) is under constitutive *ADH* promotor control. *LEU2* was used as a reporter gene. The control plate contains leucine and is used as control for equal dilution. The interaction plate lacks leucine which permits growth only to cells where AD and BD fusion proteins interact, thus enabling expression of *LEU2* gene. (b) Schematic representation of Bimolecular fluorescence complementation assay (BiFC). Proteins of interest are genetically fused to the C- and N-terminal domains of the improved YFP version Venus (VN and VC). Upon interaction of the proteins, Venus fluorescence is reconstituted. (c) Fluorescence microscopy images of BiFC analyzing the potential interaction between Blm10 and αSyn after 6 h of induction. VenusN + VenusC serves as a negative control and αSyn-VC + αSyn-VN as positive control. Scale bar = 5 μm.

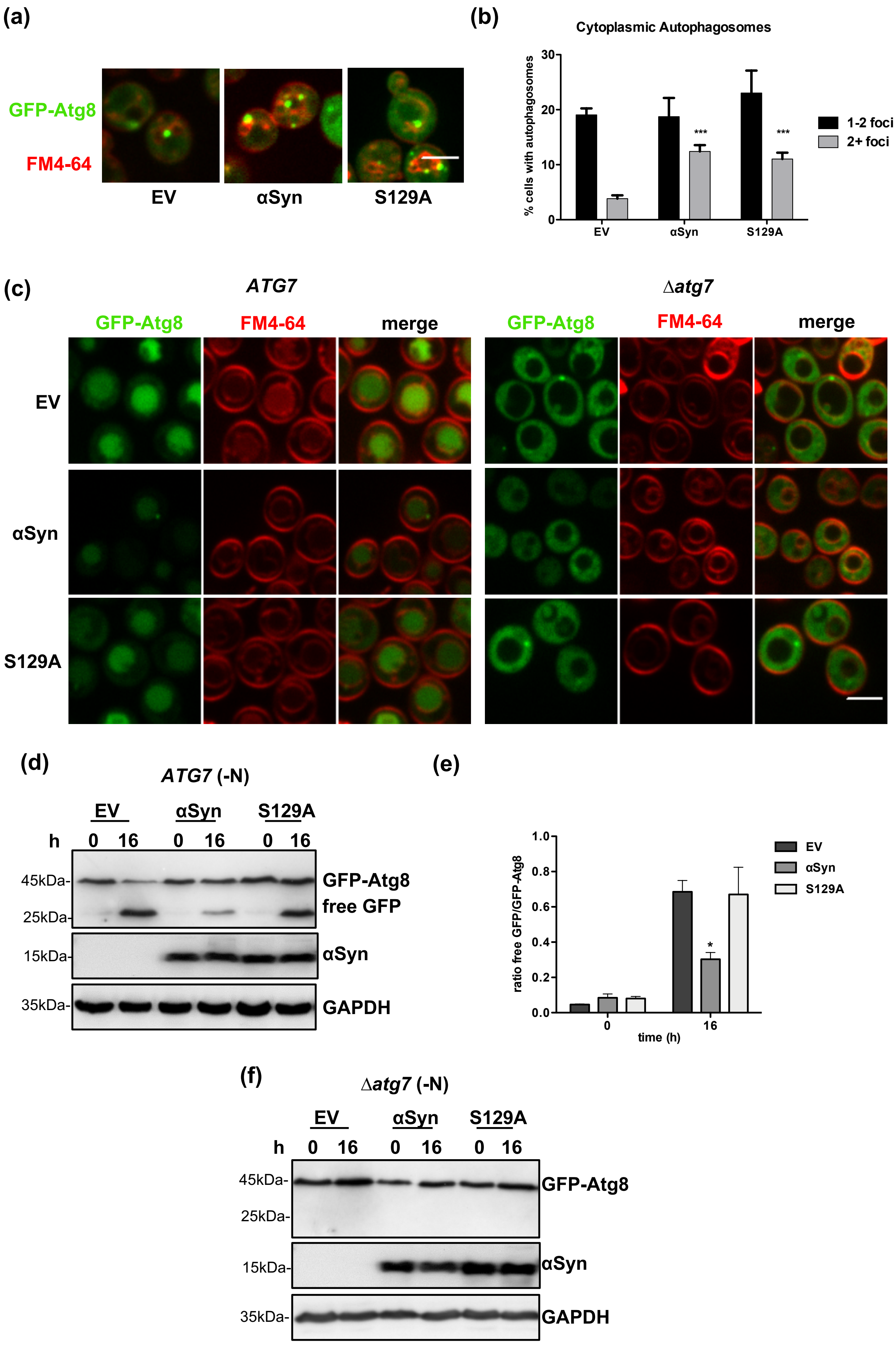

(a)
GFP-Atg8
FM4-64
EV
αSyn
S129A
(b)
Cytoplasmic Autophagosomes
% cells with autophagosomes
30
20
10
0
***
***
1-2 foci
2+ foci
EV
αSyn
S129A
(c)
ATG7
Δatg7
GFP-Atg8
FM4-64
merge
EV
αSyn
S129A
(d)
ATG7 (-N)
EV
αSyn
S129A
h
0
16
45kDa-
25kDa-
15kDa-
35kDa-
GFP-Atg8
free GFP
αSyn
GAPDH
(e)
ratio free GFP/GFP-Atg8
1.0
0.8
0.6
0.4
0.2
0.0
*
0
16
time (h)
EV
αSyn
S129A
(f)
Δatg7 (-N)
EV
αSyn
S129A
h
0
16
45kDa-
25kDa-
15kDa-
35kDa-
GFP-Atg8
αSyn
GAPDH

Figure S3. αSyn inhibits autophagy. (a) Fluorescence microcopy images of yeast cells expressing GFP-tagged Atg8 to monitor autophagosomes. FM4-64 dye was used to label the vacuolar membrane. Cells were imaged after 6 h αSyn expression. Scale bar = 5 µm. **(b)** Quantification of cells exhibiting 1-2 or more autophagosomes. Statistical significance was determined by one-way ANOVA with Dunnett's post hoc test (***p < 0.001; n = 3) in comparison to the empty vector (EV) control. **(c)** Fluorescence microscopy images of cells after 16 h of nitrogen starvation to induce autophagy. αSyn expression was induced for 6 h before induction of autophagy. GFP-Atg8 was used as an autophagy marker, with vacuolar GFP signal indicating autophagic flux. FM4-64 staining was used to visualize the vacuolar membrane. The Atg7 deletion mutant, which impairs autophagy, was used as a control. Scale bar = 5 µm. **(d)** Immunoblot analysis of yeast cells expressing GFP-Atg8 before (0 h) and after 16 h of nitrogen starvation. Autophagic flux was evaluated by detecting the release of free GFP from GFP-Atg8. αSyn expression was induced for 6 h prior to induction of autophagy. αSyn expression was conformed with αSyn antibody and GAPDH was used as a loading control. **(e)** Densitometric quantification of the ratio of free GFP to GFP-Atg8 based on scanned immunoblots. Statistical significance was determined by one-way ANOVA with Dunnett's post hoc test (*p < 0.05; n = 3) in comparison to the empty vector (EV) control. **(f)** Immunoblot analysis of yeast cells deficient in autophagy expressing GFP-Atg8, before and after 16 h of nitrogen starvation. Analysis was performed as described in (d).

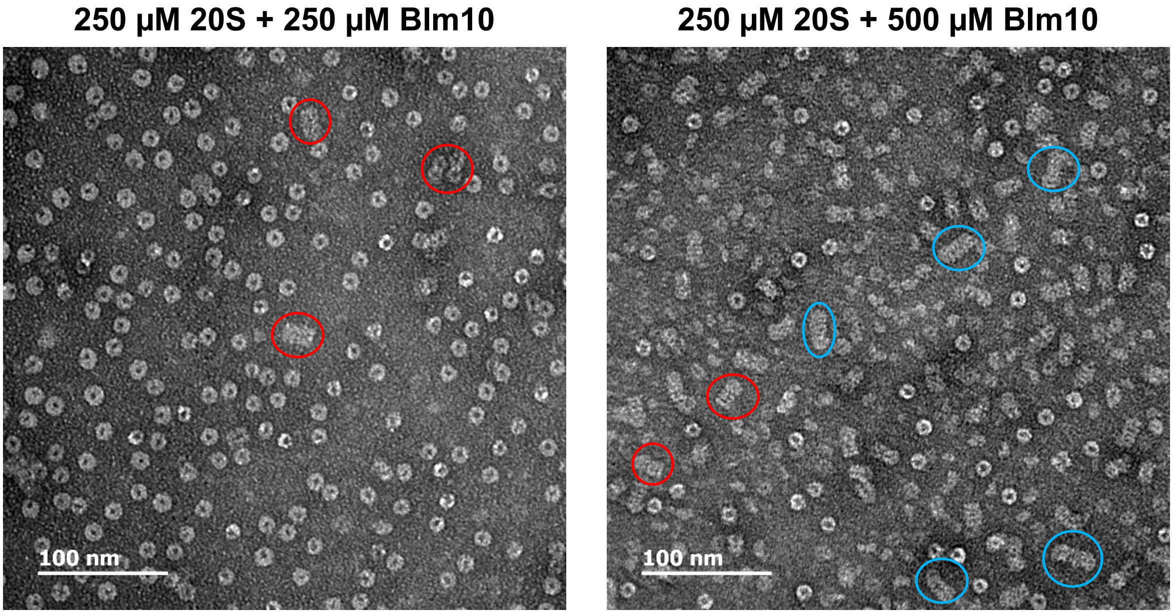

Figure S4. Reconstitution of 20S proteasomes with Blm10. Transmission electron microscopy (TEM) images of 20S proteasomes reconstituted with varying concentrations of Blm10. Reconstitution was performed by incubating purified Blm10 with purified 20S proteasome at indicated concentration for 30 min at 30 °C. Samples were applied to CF200-Cu grids, stained with 2% uranyl acetate, and imaged. Circles indicate examples of 20S proteasomes capped with one (red) or two (blue) molecules of Blm10.

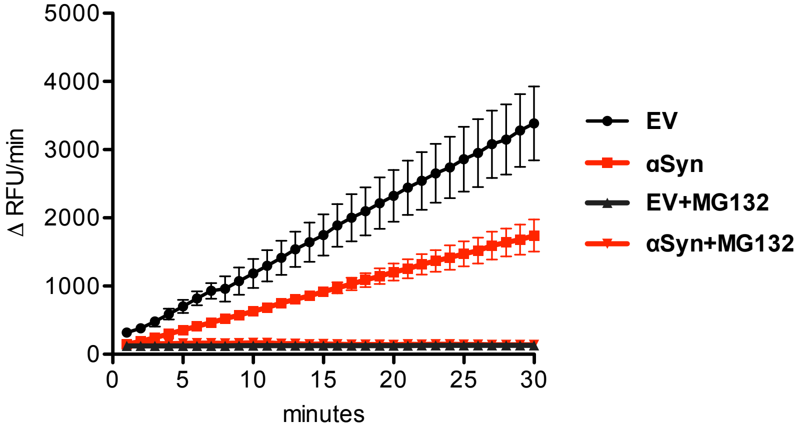

Figure S5. Proteasome activity assays with crude protein extracts. Yeast cells expressing *GAL1*-driven αSyn or with empty vector (EV) as control were collected after 6 hours of *GAL1* induction. The 26S proteasomal activity in crude protein extracts was monitored by measuring the hydrolysis of the fluorogenic peptide SUC-LLVY-AMC by detecting relative fluorescence units (RFU). As control, protein extracts from yeast cells were preincubated with 100 μM proteasome inhibitor MG132 for 10 min prior to measurement. Lack of proteasome activity is indicative for the specificity of the assay.

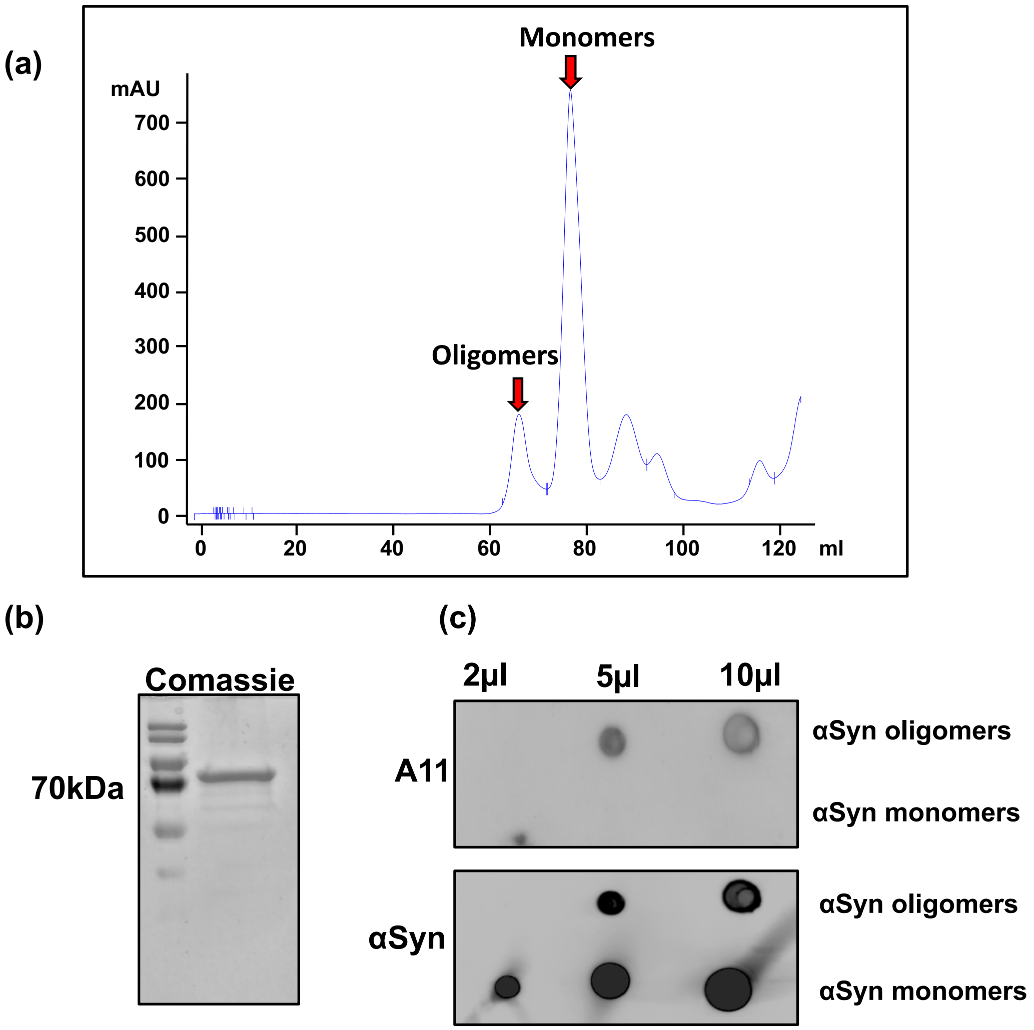

Figure S6. Purification of αSyn oligomers for activity assays. (a) Chromatogram of size exclusion chromatography (SEC) of αSyn purification from *E.coli*. Blue curve represents absorption at 280 nm. Two major peaks were defined, the first corresponds to αSyn oligomers, and the second to αSyn monomers. SEC was performed using a HiLoad 16/600 Superdex 75 column (GE Healthcare). **(b)** Comassie staining of SDS-PAGE gel of oligomer fraction following SEC. Heat and SDS-stable αSyn oligomers were detected at 70 kDa mark. **(c)** Dot blot analysis of αSyn oligomeric and monomeric fractions obtained from SEC. The A11 antibody, specific for oligomeric amyloidogenic species, was used along with αSyn antibody as a control.

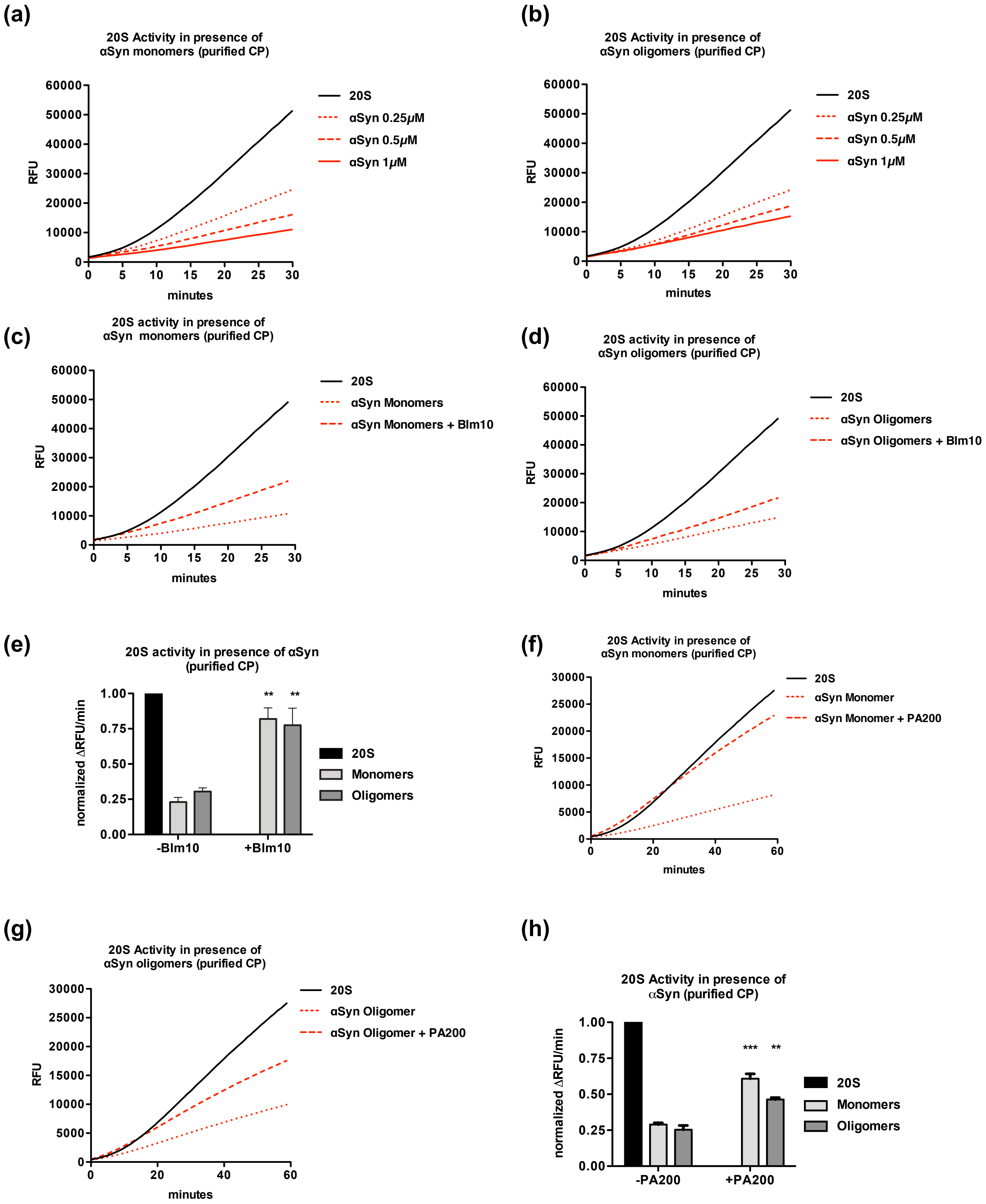

(a)
20S Activity in presence of αSyn monomers (purified CP)
RFU
minutes
20S
αSyn 0.25μM
αSyn 0.5μM
αSyn 1μM
(b)
20S Activity in presence of αSyn oligomers (purified CP)
RFU
minutes
20S
αSyn 0.25μM
αSyn 0.5μM
αSyn 1μM
(c)
20S activity in presence of αSyn monomers (purified CP)
RFU
minutes
20S
αSyn Monomers
αSyn Monomers + Blm10
(d)
20S activity in presence of αSyn oligomers (purified CP)
RFU
minutes
20S
αSyn Oligomers
αSyn Oligomers + Blm10
(e)
20S activity in presence of αSyn (purified CP)
normalized ΔRFU/min
-Blm10
+Blm10
**
**
20S
Monomers
Oligomers
(f)
20S Activity in presence of αSyn monomers (purified CP)
RFU
minutes
20S
αSyn Monomer
αSyn Monomer + PA200
(g)
20S Activity in presence of αSyn oligomers (purified CP)
RFU
minutes
20S
αSyn Oligomer
αSyn Oligomer + PA200
(h)
20S Activity in presence of αSyn (purified CP)
normalized ΔRFU/min
-PA200
+PA200
***
**
20S
Monomers
Oligomers

Figure S7. αSyn oligomers and monomers inhibit 20S proteasome activity. (a-b) SUC-LLVY-AMC activity assays of purified 20S proteasomes in the absence and presence of increasing concentrations of αSyn monomers (a) or oligomers (b). Graphs represent the mean of 3 replicates. **(c-d)** SUC-LLVY-AMC activity assays of 20S proteasomes and reconstituted 20S+Blm10 complexes in the presence of 1 μM αSyn monomers (c) or oligomers (d). Activity of 20S proteasomes alone (without Blm10 and αSyn) served as control. Data represent the mean of 3 replicates. **(e)** Mean change in fluorescence per minute calculated from (c) and (d). Statistical significance was assessed using one-way ANOVA with Dunnett's post-hoc test (**$p< 0.01$; n=3). **(f-g)** SUC-LLVY-AMC activity assays of 20S proteasomes and reconstituted 20S+PA200 complexes in the presence of 1 μM αSyn monomers (f) or oligomers (g). Activity of 20S proteasomes alone (without PA200 and αSyn) served as control. Data represent the mean of 3 replicates. **(h)** Mean change in fluorescence per minute of human 20S proteasomes with and without reconstitution with PA200 in the presence of αSyn monomers and oligomers. Statistical significance was assessed using one-way ANOVA with Dunnett's post-hoc test (***$p< 0.001$; n=3).

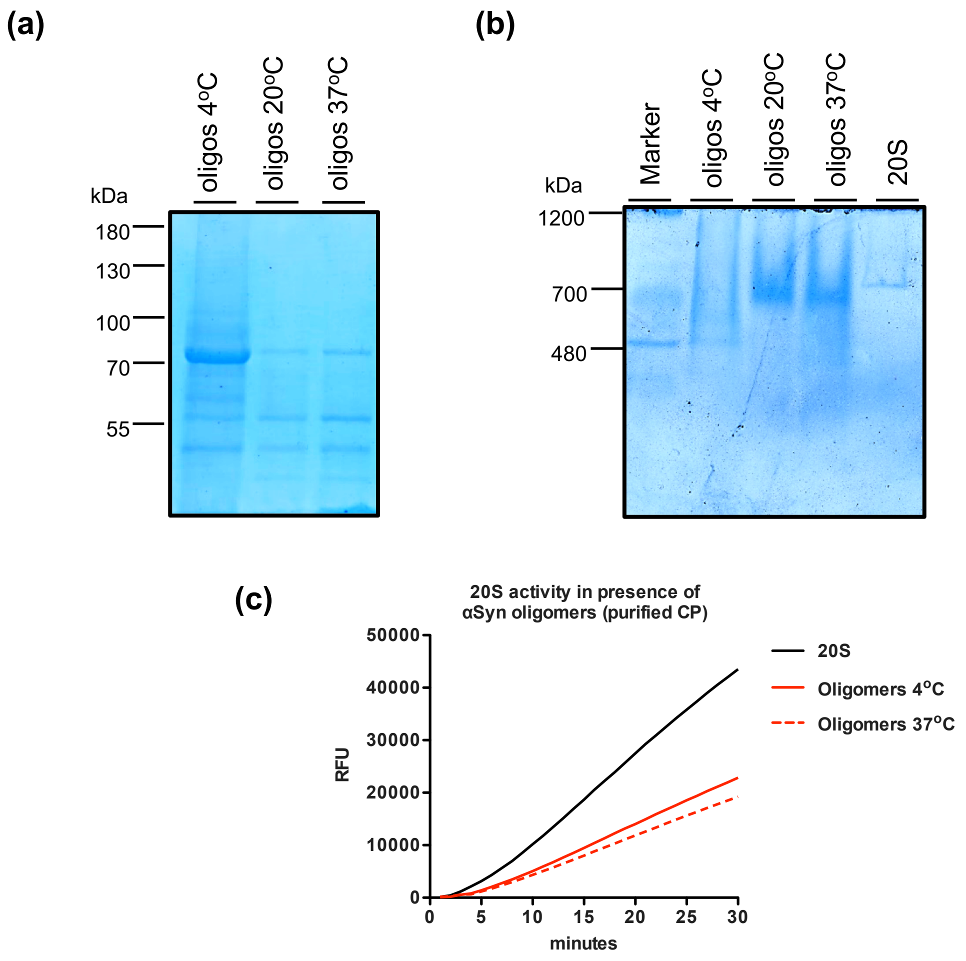

Figure S8. Characterization of oligomeric species. (a) Comassie staining of SDS-PAGE gel showing oligomeric species obtained under different conditions. Comparison of heat- and SDS-stable αSyn oligomers formed after 20 h incubation at 4ºC (corresponding to the SEC-purified oligomer fraction), 20ºC or 37ºC. **(b)** Native PAGE analysis of samples shown in (d). Purified 20S proteasomes were used as additional molecular weight marker (MW = 750 kDa). **(c)** SUC-LLVY-AMC activity assays of purified 20S proteasomes in the absence or presence of αSyn oligomeric species, formed at either 4ºC or 37ºC.
